# An Apoplastic Effector Pat-1_Cm_ of the Gram-Positive Bacterium *Clavibacter michiganensis* Acts as Both a Pathogenicity Factor and an Immunity Elicitor in Plants

**DOI:** 10.3389/fpls.2022.888290

**Published:** 2022-03-30

**Authors:** In Sun Hwang, Eom-Ji Oh, Eunbee Song, In Woong Park, Yoonyoung Lee, Kee Hoon Sohn, Doil Choi, Chang-Sik Oh

**Affiliations:** ^1^Department of Horticultural Biotechnology, College of Life Science, Kyung Hee University, Yongin, South Korea; ^2^Department of Life Sciences, Pohang University of Science and Technology, Pohang, South Korea; ^3^School of Interdisciplinary Bioscience and Bioengineering, Pohang University of Science and Technology, Pohang, South Korea; ^4^Department of Plant Science, Plant Immunity Research Center, Plant Genomics and Breeding Institute, Research Institute for Agriculture and Life Sciences, Seoul National University, Seoul, South Korea; ^5^Graduate School of Biotechnology, Kyung Hee University, Yongin, South Korea

**Keywords:** bacterial canker, catalytic triad, *Clavibacter michiganensis*, pathogenicity, serine proteases

## Abstract

*Clavibacter michiganensis*, a Gram-positive plant-pathogenic bacterium, utilizes apoplastic effectors for disease development in host plants. Here, we determine the roles of Pat-1_Cm_ (a putative serine protease) in pathogenicity and plant immunity. Pat-1_Cm_ was found to be a genuine secreted protein, and the secreted mature form did not carry the first 33 amino acids predicted to be a signal peptide (SP). The *pat-1_Cm_* mutant impaired to cause wilting, but still caused canker symptom in tomato. Moreover, this mutant failed to trigger the hypersensitive response (HR) in a nonhost *Nicotiana tabacum*. Among orthologs and paralogs of *pat-1_Cm_*, only *chp-7_Cs_* from *Clavibacter sepedonicus*, a potato pathogen, successfully complemented *pat-1_Cm_* function in pathogenicity in tomato, whereas all failed to complement *pat-1_Cm_* function in HR induction in *N. tabacum*. Based on the structural prediction, Pat-1_Cm_ carried a catalytic triad for putative serine protease, and alanine substitution of any amino acids in the triad abolished both pathogenicity and HR-inducing activities of Pat-1_Cm_ in *C. michiganensis*. Ectopic expression of *pat-1_Cm_* with an SP from tobacco secreted protein triggered HR in *N. tabacum*, but not in tomato, whereas a catalytic triad mutant failed to induce HR. Inoculation of the *pat-1_Cm_* mutant mixed with the mutant of another apoplastic effector CelA (cellulase) caused severe wilting in tomato, indicating that these two apoplastic effectors can functionally cooperate in pathogenicity. Overall, these results indicate that Pat-1_Cm_ is a distinct secreted protein carrying a functional catalytic triad for serine protease and this enzymatic activity might be critical for both pathogenicity and HR-eliciting activities of Pat-1_Cm_ in plants.

## Introduction

Plant pathogenic bacteria use diverse effector proteins to manipulate host metabolism and suppress host immunity (Dou and Zhou, 2012; Toruno et al., 2016). Effectors can be divided into intracellular and apoplastic effectors. Intracellular effectors are delivered directly into the host cell cytoplasm through type III or type VI secretion systems (T3SS or T6SS), whereas apoplastic effectors are secreted mostly through a type II secretion system (T2SS) and function in the apoplastic space of plant tissues (Costa et al., 2015; Galan and Waksman, 2018). The types and functions of intracellular effector proteins have been well studied in Gram-negative bacteria such as *Pseudomonas syringae* and *Xanthomonas* spp. Generally, these effectors act as virulence factors in susceptible host plants (Cunnac et al., 2009; White et al., 2009; Lindeberg et al., 2012; Timilsina et al., 2020). However, some effectors act as avirulence factors and are recognized by the Nod-like receptor (NLR) proteins to trigger an immune response in nonhost and resistant plants (Lee et al., 2017; van Wersch et al., 2020). In contrast, apoplastic effectors such as cell wall-degrading enzymes, cellulases and pectate lyases, and proteases have been studied in soft rot pathogens (Davidsson et al., 2013) and have been shown only as virulence factors. Unlike soft rot pathogens, *Cladosporium fulvum*, a fungal pathogen that causes leaf mold disease in tomato, uses apoplastic effectors such as Avr2, Avr4, and Avr9 as virulence factors to suppress pattern-triggered immunity and can trigger immune responses upon recognition by plasma membrane-localized resistance proteins, Cf (de Wit, 2016).

Unlike Gram-negative bacteria, the types and functions of effector proteins have not been well studied in Gram-positive plant-pathogenic bacteria. Nevertheless, some apoplastic effectors have been studied as major virulence factors. As an example, a Gram-positive phytopathogenic bacterium, *Clavibacter michiganensis*, causes bacterial canker and wilting in tomato and uses a cellulase CelA, a pectate lyase PelA, and serine proteases such as Pat-1_Cm_ and the Chp (Chromosomal homologs of Pat-1) protein family as major virulence factors (Eichenlaub and Gartemann, 2011; Thapa et al., 2017; Hwang et al., 2018, 2019). Meanwhile, other Gram-positive bacteria such as *Streptomyces scabies*, which causes scab in potato tuber, and *Rhodococcus fascians*, which causes the leafy gall disease, use the phytotoxin, thaxtomin, and phytohormones as major pathogenicity or virulence factors, respectively (Bignell et al., 2010; Stes et al., 2013; Francis et al., 2016; Liu et al., 2021).

*Clavibacter michiganensis* Pat-1_Cm_ is a putative serine protease encoded by the *pat-1* gene in the pCM2 plasmid. It carries a putative signal peptide (SP) in its N-terminus (Lu et al., 2015), indicating that Pat-1_Cm_ may be secreted. Observations of a *C. michiganensis* strain lacking pCM2 and complementation of this strain with a DNA fragment carrying the *pat-1_Cm_* gene implied that the *pat-1_Cm_* gene functions as a pathogenicity factor, although there were no experimental data using deletion or defective mutants of the *pat-1_Cm_* gene (Dreier et al., 1997; Burger et al., 2005). Although the enzymatic activity of these proteins has not been demonstrated yet, Pat-1_Cm_ and Chp family proteins have a shared serine residue within a conserved GDSGG motif that might be one of a catalytic triad (together with histidine and aspartate) for protease activity (Ruiz-Perez and Nataro, 2014; Lu et al., 2015). A Pat-1 ortholog in *Clavibacter sepedonicus*, Chp-7_Cs_ was found to be involved in virulence in potato and hypersensitive response (HR) induction in *Nicotiana tabacum* (Nissinen et al., 2009), indicating that Pat-1_Cm_ and its orthologs might act as both pathogenicity or virulence factors and immunity elicitors in plants. Moreover, the analysis of whole genome sequence data of *Clavibacter* species has shown that many genes encoding these putative serine proteases with SP are present in various *Clavibacter* species, including *C. michiganensis*, *Clavibacter capsici*, and *C. sepedonicus* (Nissinen et al., 2009; Lu et al., 2015; Hwang et al., 2018; Mendez et al., 2020). Overall, these findings suggest that Pat-1_Cm_ and its orthologs are important apoplastic effectors for the interaction of *Clavibacter* species with plants.

Similar cases have been shown in other pathogenic bacteria. The secreted serine protease, PrtA, of *Xylella fastidiosa* and a cysteine protease HopN1 of the YopT/AvrPphB effector family in *P. syringae* pv *tomato* DC3000 contribute to the virulence and HR induction, respectively (Shao et al., 2002; Gouran et al., 2016). However, unlike human and animal pathogens (Ruiz-Perez and Nataro, 2014), the role of serine proteases in virulence of phytopathogenic bacteria is still elusive, especially in Gram-positive bacterial pathogens such as genus *Clavibacter*.

In this study, we characterized Pat-1_Cm_ with respect to its protein secretion, pathogenicity, and ability to induce HR in plants. We found that Pat-1_Cm_ is indeed a secreted protein with a functional SP and has roles in pathogenicity and plant immunity. Moreover, the catalytic triad of Pat-1_Cm_ for putative serine protease is critical for both its pathogenicity and HR induction in plants. Our findings provide us a distinct role of apoplastic protease effectors of pathogenic bacteria for interactions with host and nonhost plants.

## Materials and Methods

### Bacterial Strains, Culture Conditions, and Inoculum Preparation

The *C. michiganensis* type strain LMG7333, its mutant strains, Tn::*pat-1_Cm_* and Tn::*celA_Cm_* generated by transposon mutagenesis, and the complemented strains were used in this study (Hwang et al., 2019). All bacterial strains were grown on KB medium (20 g of protease peptone no. 3, 1.5 g of K_2_HPO_4_, 6 ml of 1 M MgSO_4_, and 16 ml of 50% glycerol per liter) supplemented with appropriate antibiotics: kanamycin (100 μg ml^−1^), neomycin (100 μg ml^−1^), and chloramphenicol (10 μg ml^−1^) at 26°C for 24–48 h. For plant inoculation, single colonies of cultured *C. michiganensis* strains were incubated in KB broth overnight with shaking at 140 rpm, and then cells were collected by centrifugation and resuspended in 10 mM of MgCl_2_.

### Plant Growth Conditions

*Tomato* (*Solanum lycopersicum* L., cv. “*Betatini*”) and *N. tabacum* (cv. “*Samsun*”) plants were grown in a growth chamber at 26°C with a 14:10-h light:dark photoperiod condition. Then, 2- or 3-week-old tomato plants and 6-week-old *N. tabacum* plants were used for disease and HR assays, respectively.

### Selection of Tn::*pat-1_Cm_* Mutant

To select a mutant with a transposon insertion in the *pat-1_Cm_* gene, we screened approximately 1,400 *C. michiganensis* strain LMG7333 mutants generated with a transposon, Tn*1409*Cβ, in the vector pKGT452Cβ (Kirchner et al., 2001; Hwang et al., 2019) by virulence assay in tomato and by PCR using specific primer set (Hwang et al., 2019). The transposon insertion site was determined by whole genome sequencing of the mutant using Illumina sequencing and genome comparison with the genome sequence of the WT *C. michiganensis* strain LMG7333 (GenBank accession nos. CP080437, CP080438, and CP080439). Furthermore, the single transposon insertion was confirmed by southern hybridization using the chloramphenicol resistance gene as a probe.

### Plasmid Curing in *Clavibacter michiganensis* LMG7333

To remove the large plasmid (pCM2) carrying the *pat-1_Cm_* gene, the WT *C. michiganensis* strain LMG7333 was incubated at 26°C for 2 days and moved to 37–42°C for 3 days. This temperature change was repeated several times in a fresh medium, and then the bacterial culture was spread on KB plates. To check the presence of pCM2, colonies grown on KB plate were screened by PCR using primer sets targeting plasmid backbone genes and the *pat-1_Cm_* gene (Supplementary Table 1). The pCM2-cured strain, LMG7333ΔpCM2, was selected and used for this study.

### Virulence and HR Assays in Plants

Two-week-old tomato seedlings were inoculated with *C. michiganensis* strains by the root-dipping inoculation method described by Hwang et al. (2019). Inoculums were prepared from freshly cultured bacteria, and their concentration was adjusted to OD_600_ = 2.0 [1 × 10^9^ colony forming units (CFU) ml^−1^] with 10 mM MgCl_2_. Whole tomato seedlings were pulled out of the soil, and, if necessary, trimmed with sterile scissors. Then, the prepared seedlings were submerged into tubes containing 1 ml of bacterial inoculum for 30 min. As a negative control (mock), seedlings were inoculated with 10 mM MgCl_2_. Inoculated seedlings were transplanted into soil again in mini pots and grown in a growth chamber at 26°C. Approximately 10–14 days after inoculation (dai), the disease severity of above-ground disease symptoms of all seedlings was evaluated. Wilting symptom severity for each seedling was rated from 0 to 5 scales based on disease index defined by Hwang et al. (2019), which is as follows: 0, no visible symptoms; 1, one or two leaves mildly wilted; 2, more than two leaves mildly wilted, but less than two leaves severely wilted; 3, 25–50% of leaves severely wilted; 4, 50–75% of leaves severely wilted or dead; and 5, all leaves severely wilted or dead. All experiments were performed at least three times with 10 plants per treatment (*n* = 10). For stem inoculation, the stems of 3-week-old tomato plants were wounded above the cotyledons, the wounds were inoculated with 10 μl of *C. michiganensis* inoculum, and then the infected plants were transferred to a growth chamber for 3 weeks for canker development.

For the HR assay, leaves of 6-week-old *N. tabacum*, a nonhost plant, were used. Each *C. michiganensis* strain inoculum, adjusted to a concentration of OD_600_ = 0.05, was infiltrated by needless syringe into 6-week-old *N. tabacum* plant leaves. HR development in the infiltrated leaves was observed for 48 h.

### *Agrobacterium*-Mediated Transient Expression Assay in *Nicotiana tabacum* Leaves

The ORFs of *pat-1*_Cm_, *chpC*_Cm_, *chpE*_Cm_, *chpF*_Cm_, and *chpG*_Cm_ genes without the stop codon were amplified from genomic DNA of *C. michiganensis* strain LMG7333 (Supplementary Table 1). The amplified DNA fragments were cloned into the pENTR/SD/D-TOPO vector (Invitrogen, CA, United States) according to the manufacturer’s instructions. After cloning, a gene fragment encoding a signal peptide of tobacco PR1b (GenBank accession no. X03465.1) was inserted into their N-terminal sites to generate in-frame fusion proteins and was verified by DNA sequencing. The resulting entry clones were recombined with the Gateway pGWB417 destination vector by LR reactions (Nakagawa et al., 2007). All cloned genes were also fused with c-Myc tag in their C-termini and expressed under the control of the 35S promoter. Recombinant plasmid constructs were transformed into *Agrobacterium tumefaciens* strain GV3101 for further assay.

For transient expression assay in *N. tabacum* leaves, *A. tumefaciens* strains with target genes were grown overnight in YEP medium (10 g of yeast extract, 1 0 g of peptone, and 5 g of NaCl per liter) with rifampicin (50 μg ml^−1^) and spectinomycin (50 μg ml^−1^) at 26°C. *Agrobacterium* cells were collected by centrifugation, washed with infiltration buffer (10 mM MES and 10 mM MgCl_2_), and resuspended in the same buffer. Bacterial suspension with 100 μM acetosyringone was incubated at 25°C for more than 3 h and centrifuged again. After adding the infiltration buffer with 100 μM acetosyringone, the bacterial suspension was adjusted to an OD_600_ of 0.4. Leaves of 6-week-old *N. tabacum* plants were infiltrated with an *Agrobacterium* suspension using a needleless syringe, and the plants were kept in the light to dry the leaves and subsequently incubated at 25°C.

### Ion Conductivity Measurement

To measure electrolyte leakage to quantify the degree of HR development, a total of six leaf disks (9.2 mm diameter) were harvested from infiltrated leaves at 0 and 24 hai. Detached leaf disks were washed with 20 ml of deionized water for 30 min and then placed in a new tube containing 20 ml of deionized water and shaken at 160 rpm for 2 h. Ion conductivity was measured at the indicated time points with a conductometer (*CON6* portable conductivity meter; *Oakton*, IL, United States).

### Generation of *pat-1* Gene Constructs

The *pat-1*_Cm_ gene and its variants, including an SP-deleted mutant and six alanine-substituted mutants with 0.4-kb native promoter region and a FLAG tag on their C-termini were amplified from *C. michiganensis* strain LMG7333 by PCR and cloned into the pTOP blunt V2 vector (Enzynomics, Daejeon, Korea), named pTOP-*pat-1*_Cm_. Moreover, to generate Tn::*pat-1_Cm_* strains in which *pat-1_Cm_* orthologous genes present in *C. capsici* and *C. sepedonicus* were expressed under the native *pat-1*_Cm_ gene promoter region, *pat-1_Cc_* was amplified from *C. capsici* strain PF008 by PCR. The *pat-1_Cs_* and the paralogous *chp-7_Cs_* gene from *C. sepedonicus* were obtained by custom gene synthesis with the promoter region of the *pat-1*_Cm_ gene (Bioneer, Daejeon, Korea). The DNA fragments of all *pat-1* genes digested by *Spe*I/*Hind*III restriction enzymes were ligated into the linearized pK2-22 vector with the same enzymes.

To generate Pat-1_Cm_ variants with alanine substitutions at specific amino acid residues, amino acid substitutions of *pat-1_Cm_* were performed by site-directed mutagenesis. Using the construct pTOP-*pat-1*_Cm_ as a template, PCR was performed with two mutagenic primers and then treated with *Dpn*I to digest the methylated template. The final product was transformed into *Escherichia coli* strain DH5α and then confirmed by PCR and DNA sequencing. These variant genes were cloned into the pK2-22 vector.

Bacterial complementation using the pK2-22 vector was performed as previously described (Hwang et al., 2018). Briefly, the mutant Tn::*pat-1_Cm_* was transformed with each gene construct, in which a FLAG tag was fused at the C-terminus, into the pK2-22 vector. After transformation by electroporation with up to 4–5 μg of plasmid DNA, the transformants were grown over 3 days at 26°C, and then the single transformed bacterial colony was selected with neomycin (50 μg ml^−1^) as a selectable marker.

### Western Blotting and Proteomics Analysis

Bacterial strains containing C-terminal FLAG-tagged gene constructs were grown in half-strength KB media with 0.4% CMC. Incubated bacterial cells were harvested by centrifugation, and its supernatant was precipitated with 10% w/v of chilled trichloroacetic acid (TCA)/acetone at a ratio of 4:1 after filtration with a 0.22 μm pore size sterile *filter*. Proteins collected in the supernatant by centrifugation were washed twice with ice-cold acetone and resuspended in 0.1 ml distilled water. Proteins from the cell pellets were lysed at 25°C for 20 min using *B*-P*ER™ Bacterial* Protein *Extraction Reagent* (Thermo Scientific, Rockford, IL, United States) supplemented with 100 μg ml^−1^ lysozyme. After centrifugation, the supernatant extracted from the cell pellet was used for western blot analysis.

For total protein extraction from *N. tabacum* leaves, inoculated leaves were harvested at 36 hai and ground into a fine powder in liquid nitrogen. Samples were homogenized in protein extraction buffer [10% glycerol, 150 mM Tris–HCl (pH 7.5), 1 mM EDTA, 150 mM NaCl, 5 mM dithiothreitol, protease inhibitor cocktail (Sigma, St. Louis, MO, United States), and 0.2% Triton X-100 (Sigma)]. The homogenates were centrifuged at 12,000 *g* for 20 min at 4°C, and supernatants were collected for further study.

For western blot analysis, protein samples in sodium dodecyl sulfate (SDS) sample buffer [100 ml of 1.5 M Tris (pH 6.8), 60 ml of 20% SDS, 300 ml of glycerol, 150 ml of β-mercaptoethanol, and 18 mg of bromophenol blue per liter] were denatured by boiling and separated in a 12% SDS-polyacrylamide gel at 100 V for 2–3 h. After electrophoresis, separated proteins were transferred onto a polyvinylidene difluoride membrane (Millipore, Burlington, MA, United States). Subsequently, blots were blocked for 1 h with 5% w/v nonfat milk in phosphate-buffered saline (PBS) with 0.1% w/v Tween 20 and immunoblotted with horseradish peroxidase-conjugated anti-FLAG antibody (Sigma-Aldrich, St. Louis, MO, United States) or anti-c-Myc antibody (Santa Cruz Biotechnology, Santa Cruz, CA, United States), followed by anti-mouse IgG-HRP secondary antibody (Santa Cruz Biotechnology). Proteins were detected by the HRP activity using enhanced chemiluminescence plus western blotting detection reagent (GE Healthcare, Little Chalfont, United Kingdom).

For 2D gel electrophoresis, proteins were precipitated from cell-free supernatant of *C. michiganensis* type strain LMG7333 by 10% TCA-acetone precipitation. Isoelectric focusing was performed using 24 cm immobilized pH gradient strips with a non-linear pH 4–10 gradient. 2D gel electrophoresis and MALDI-TOF MS analysis were conducted at GenoMine (Pohang, Gyeongbuk, Korea).

### Protein Structure Prediction

A tertiary structure of the Pat-1_Cm_ protein was predicted using phyre2^1^ based on the principles of homology-based modeling (Kelley et al., 2015).

### Purification of Mature Pat-1_Cm_

To obtain a large amount of Pat-1_Cm_ proteins tagged with C-terminal FLAG, *C. michiganensis* Tn::*pat-1_Cm_* expressing *pat-1_cm_* or its variants was cultured in 1/5 strength KB media with 0.2% CMC for 2 days at 26°C with constant shaking. The large-scale cultures (500 ml) were centrifuged to collect the supernatant, and then the supernatant was filtered through a 0.22 μm sterile filter (Sartorius Stedim Biotech GmbH, Göttingen, Germany). To immune-precipitate *Pat-1_Cm_* protein, 300 μl of washed Pierce Anti-DYKDDDDK Magnetic Agarose (A36797; Thermo Scientific) was added to 50 ml of filtered supernatant and incubated with rotation for 1 h. The tube with the anti-FLAG magnetic beads/protein was placed on a magnetic stand, and the supernatant was removed. This procedure was repeated several times to collect enough protein for the experiment. After removing the supernatant, bound beads were washed twice with PBS (pH 7.4) and once with distilled water. Washed beads were eluted with Pierce IgG Elution buffer (pH 2.8, 21004; Thermo Scientific) or SDS-PAGE sample buffer.

### N-Terminal Sequencing of Mature and Secreted Pat-1_Cm_

To determine the N-terminal amino acids of mature and secreted Pat-1_Cm_, purified proteins extracted from cell-free supernatant were separated by 1D SDS-PAGE and blotted onto PVDF (Immobilon-P membrane, Merck Millipore, MA, United States). After electro-blotting, the membrane was stained with Coomassie Blue R-250 for 30 min, de-stained multiple times with de-staining solution (10% acetone and 45% methanol–water per liter), and then rinsed with distilled water to remove the high concentration of other buffers, including transfer buffers. This membrane was dried at 25°C, and the targeted protein bands were excised with a blade. For N-terminal sequencing, the cut membrane was subjected to automated Edman degradation using a Procise 492 Protein Sequencing System (Applied Biosystems, CA, United States) at PROTEINWORKS (Daejeon, Korea).

### Statistical Analysis

Statistical analysis of disease severity data was done by applying a non-parametric Kruskal-Wallis test with Dunnett’s multiple comparisons (*p* < 0.01) using the software statistiXL version 2.0 (statistiXL, Broadway, Australia). Duncan’s multiple range test was performed to analyze other results for comparisons between independent groups (*p* < 0.05).

## Results

### *Clavibacter michiganensis pat-1_Cm_* Gene Is Critical for the Development of Wilting Symptoms, but Not Canker Symptoms, in Tomato

Previously, the curing of plasmid pCM2 and complementation analysis with the *pat-1_Cm_* gene in pCM2 showed that the *pat-1_Cm_* gene is critical for wilting caused by *C. michiganensis* strain NCPPB382 in tomato (Meletzus et al., 1993; Dreier et al., 1997). To further study the role of the *pat-1_Cm_* gene as a pathogenicity factor, we screened a *C. michiganensis* type strain LMG7333 mutant library generated by transposon (Tn*1409*Cβ) insertion (Hwang et al., 2019) and selected the LMG733-Tn::*pat-1_Cm_* mutant strain (hereafter Tn::*pat-1_Cm_* strain; Supplementary Figure 1). The transposon’s position was determined by whole genome sequencing using Illumina MiSeq. The transposon was located at a distance of 542 bp from the translation start site (ATG) of *pat-1_Cm_* (Supplementary Figure 1A), and a single insertion was confirmed by Southern blot analysis (Supplementary Figure 1B). Simultaneously, we generated the pCM2-curred strain (7333ΔpCM2) by plasmid curing. Transposon insertion and pCM2 absence were confirmed by PCR with primer pairs targeting *pat-1_Cm_* and the plasmid backbone gene within pCM2 (Supplementary Figure 1C).

We next determined the pathogenicity of the Tn::*pat-1_Cm_* strain and LMG7333ΔpCM2 for wilting symptoms in 2-week-old tomato plants *via* the root-dipping inoculation method and for bacterial canker symptom in 3-week-old tomato plants *via* the stem-inoculation method. The wild-type (WT) LMG7333 strain caused severe wilting that led to the death of inoculated plants (Supplementary Figure 1D). However, tomato plants inoculated with either Tn::*pat-1_Cm_* strain or 7333ΔpCM2 strain exhibited only very mild or no wilting symptoms (Supplementary Figure 1D), although overall plant growth was slightly reduced after infection with both mutant strains. Interestingly, the *C. michiganensis* WT and its two mutants caused a similar degree of canker symptoms around the inoculation sites (Supplementary Figure 1E). When the stems of the WT-inoculated plants showing canker symptoms were cut lengthwise and examined, the brown discoloration in the vascular tissues and the tissue collapse typical of severe wilting symptoms were observed (Supplementary Figure 2). In contrast, the decrease in discoloration of vascular tissues was only observed in the absence of tissue collapse and of wilting symptoms in plants inoculated with the Tn::*pat-1_Cm_* strain (Supplementary Figure 2).

**Figure 1:**
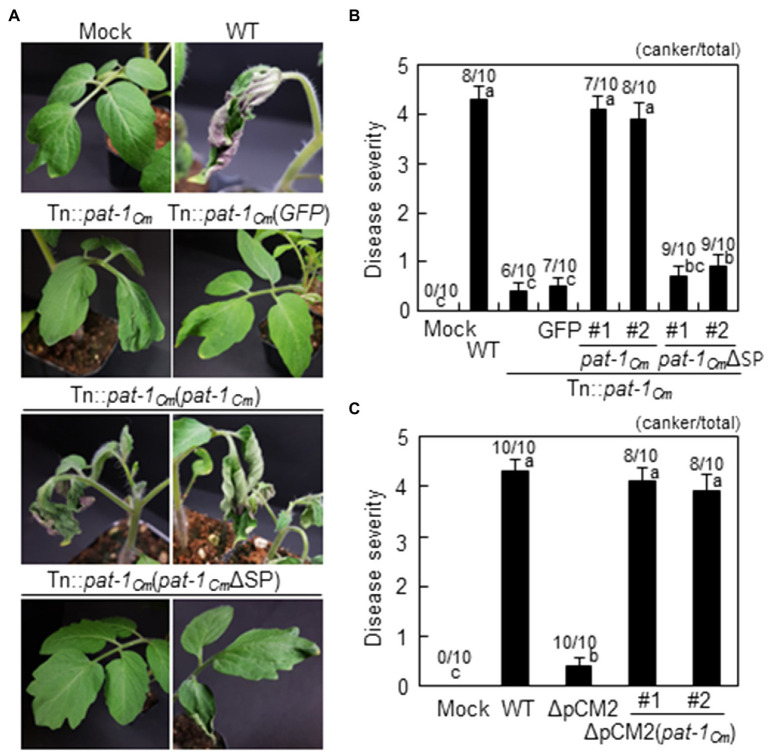
Pathogenicity recovery of *Clavibacter michiganensis* mutant Tn::*pat-1_Cm_* transformed with intact *pat-1_Cm_* gene, but not *pat-1_Cm_*ΔSP, in tomato. **(A)** Disease symptom development in infected tomato plants with indicated strains. Complemented strains was generated by overexpressing either full-length *pat-1_Cm_* or signal peptide (SP)-lacking *pat-1_Cm_*ΔSP. Two-week-old plants were used, and disease symptoms were photographed 14 days after inoculation (dai). **(B,C)** Disease severity of wilting in tomato plants inoculated with the indicated strains at 14 dai calculated based on disease index. Moreover, plants with bacterial canker were marked as the number of plants that displayed canker on the stem (canker/total). Error bars indicate SE (*n* = 10). Nonparametric Kruskal–Wallis test with Dunnett’s multiple comparisons (*p* < 0.05) was used to analyze the level of disease severity in tomato plants, and different letters indicate statistically significant differences at *p* < 0.05. C. Mock, 10 mM MgCl_2_; WT, *C. michiganensis* LMG7333 wild–type (WT).

The mutant strains were complemented by transformation with the intact *pat-1_Cm_* gene controlled by its native promoter and fused with a FLAG tag at its 3′-terminus, named Tn::*pat-1_Cm_* (*pat-1_Cm_*) and 7333ΔpCM2 (*pat-1_Cm_*). The ability to cause wilting in tomato as much as the WT was restored in both complemented strains (Figure 1; Supplementary Figure 3), indicating that the *pat-1_Cm_* gene is a critical factor for wilting in the host plant, tomato. Nevertheless, all strains, including mutants and the complementary strains, were consistently able to cause bacterial canker on plant stems (Supplementary Figures 1E, 2). Overall, these results indicate that *pat-1_Cm_* in the plasmid pCM2 is critical for *C. michiganensis* to cause wilting, but not canker, in tomato.

### The Pat-1_Cm_ Protein Carries a Functional SP and Is Secreted

**Figure 2:**
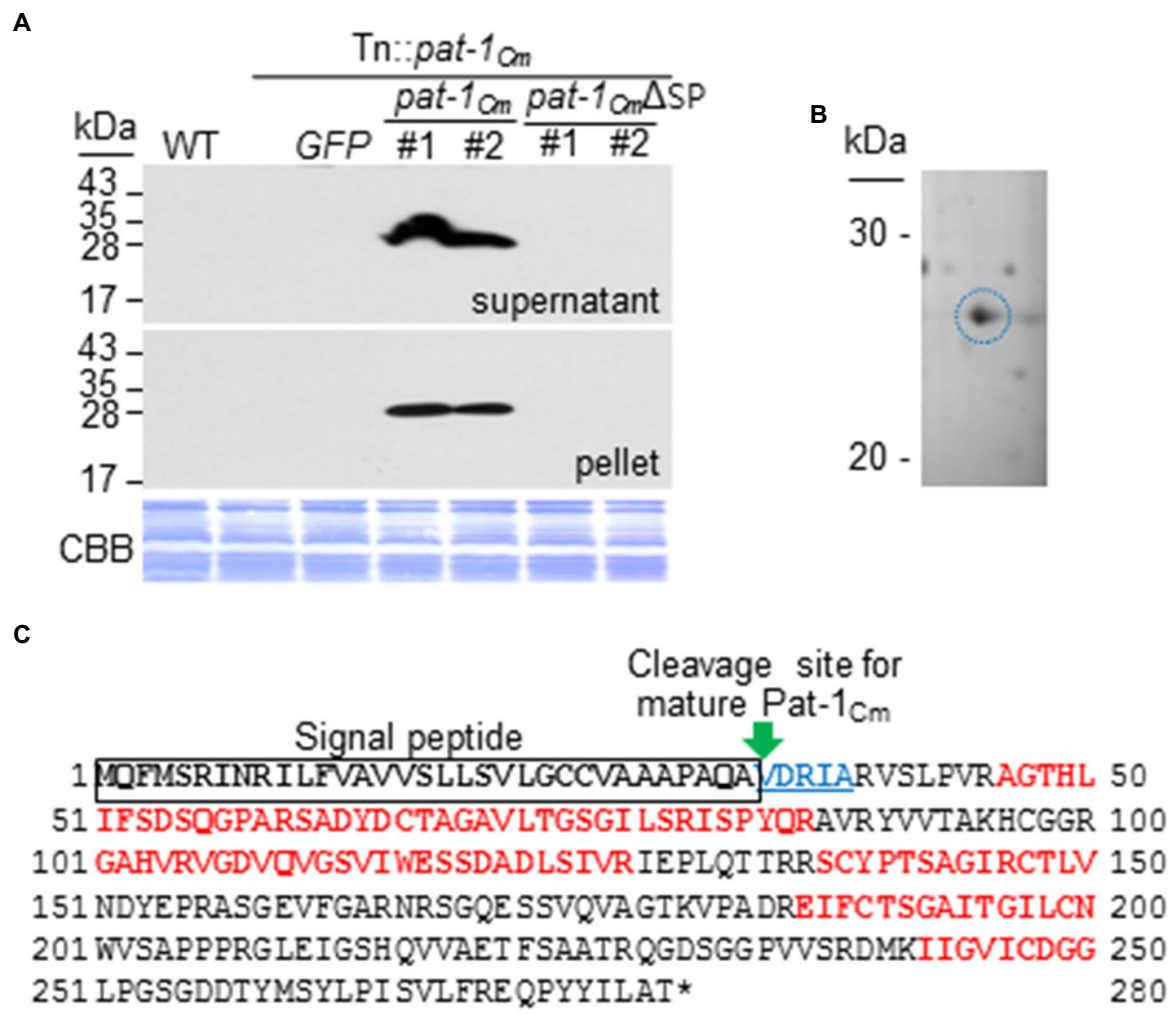
Pat-1_Cm_ proteins are secreted and well-expressed in complementary strains. **(A)** Expression and secretion of Pat-1_Cm_ proteins in complemented strains by western blotting. Pat-1_Cm_ protein and its derivative were fused to FLAG on their C-termini and expressed in Tn::*pat-1_Cm_* strain. Total proteins from the supernatant and pellet were analyzed by immunoblotting using the FLAG antibody. CBB, Coomassie Brilliant Blue. Mock, 10 mM MgCl_2_; WT, *Clavibacter michiganensis* LMG7333 wild type. **(B)** Pat-1_Cm_ proteins secreted in supernatant and separated by 2D gel. Pat-1_Cm_ protein spot (blue circle) was analyzed by mass spectrometry (see Supplementary Figure 2C). **(C)** Identification of Pat-1_Cm_ proteins by 2D-LC MS/MS. Red bolded letters indicate peptide sequences obtained from mass spectrometry. Black bolded letters indicate predicted SP sequence. Five blue bolded underlined letters indicate N-terminal sequences of mature Pat-1_Cm_ obtained by Edman degradation.

The *pat-1_Cm_* gene of the *C. michiganensis* type strain LMG7333 encodes a protein consisting of 280 amino acids that harbors a putative SP in the N-terminal region (Supplementary Figure 4A). Additional protein comparison revealed that Pat-1 proteins from various *Clavibacter* species contain a conserved protein sequence, including three amino acids for a catalytic triad of putative serine proteases and two cysteines. This holds true even with the Pat-1_Cs_ protein from *C. sepedonicus*, which lacks an SP. Since Pat-1 was predicted to have an SP in its N-terminal region, we hypothesized that Pat-1*_Cm_* is a secreted protein with a functional SP. To ensure the importance of Pat-1*_Cm_* secretion for *C. michiganensis* pathogenicity, the first 33 amino acids, predicted as an SP, were removed, and the resulting truncated form, *pat-1_Cm_*ΔSP was tagged with FLAG at its C-terminus and transformed into the Tn::*pat-1_Cm_* strain. This complementary strain did not cause wilting in tomato (Figures 1A,B). Immunoblot with a polyclonal FLAG antibody detected the FLAG-tagged full-length of Pat-1*_Cm_* from both the supernatant and pellet after growth of both complemented strains in King’s B (KB) medium with 0.4% carboxymethyl cellulose (CMC) as a substrate (Figure 2A). However, the Pat-1*_Cm_*ΔSP in the Tn::*pat-1_Cm_* strain was not detected in either fraction. These results indicate that Pat-1_Cm_ protein is expressed in the complemented strains and that the predicted SP is required for secretion and might be critical for protein stability.

To reconfirm that Pat-1_Cm_ proteins are secreted, *C. michiganensis* strain LMG7333 was first grown in KB medium with 0.4% CMC and pelleted, and then the cell-free supernatant was collected. The total protein content of the cell-free supernatant was separated by 2D gel electrophoresis, and distinct protein spots were analyzed by MALDI TOF MS/MS. As a result, Pat-1_Cm_ protein was detected (Figure 2A) and possessed a 100% match to five peptide sequences (Figure 2B).

To determine the SP cleavage site of this protein, we analyzed the N-terminal sequences of mature and secreted Pat-1*_Cm_* using Edman degradation. The first five amino acid residues (V-D-R-I-A) were obtained by N-terminal sequencing (Figure 2C; Supplementary Figure 5). These results indicate that Pat-1*_Cm_* is a genuine secreted protein with the functional SP consisting of the first 33 amino acids.

### The *pat-1_Cm_* Gene Is Required for HR Induction in a Nonhost Plant

**Figure 3:**
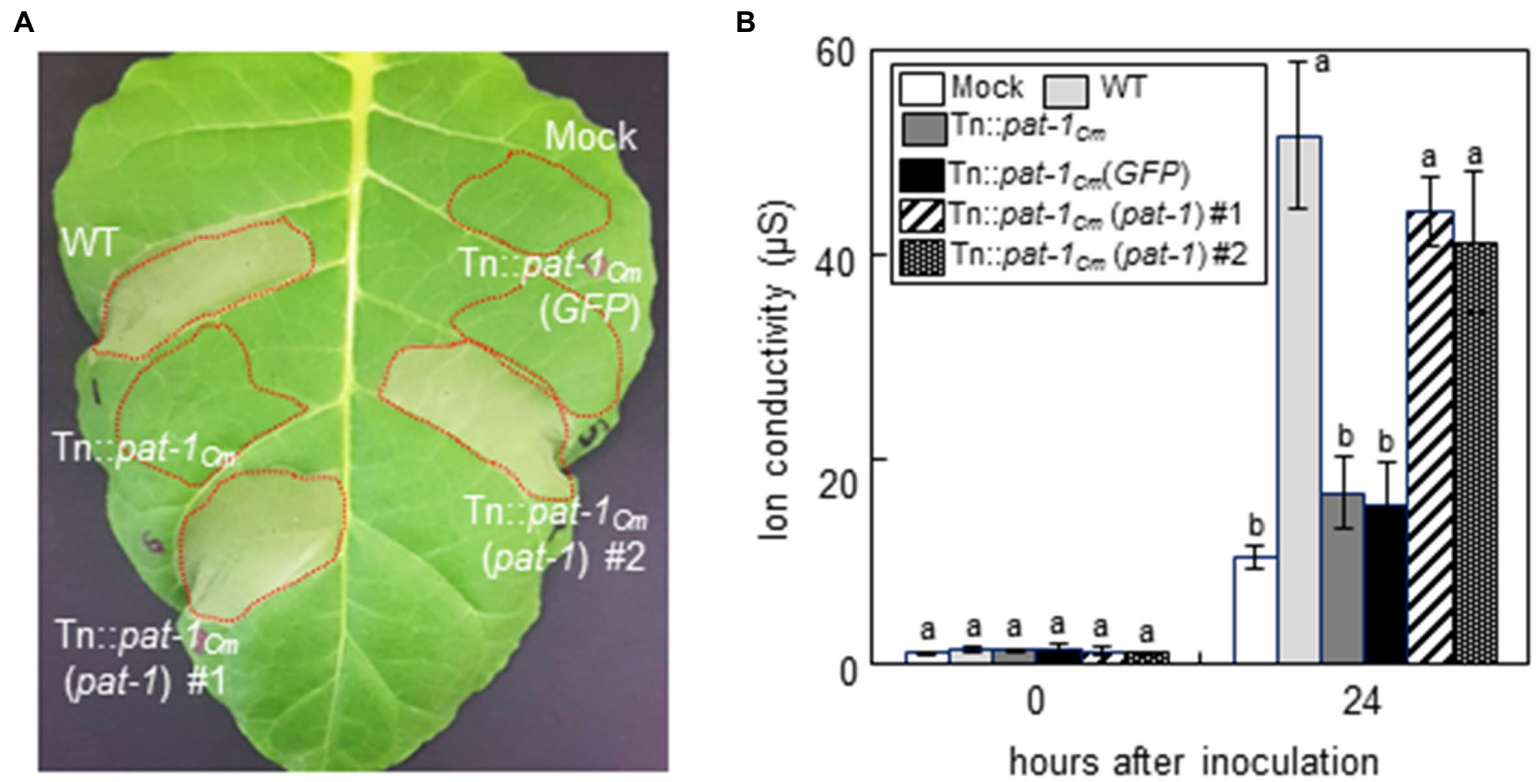
Loss of hypersensitive response (HR)-eliciting activity of *Clavibacter michiganensis* mutant Tn::*pat-1_Cm_* in a nonhost plant, *Nicotiana tabacum*. **(A)** HR phenotype in *N. tabacum* after infiltration with the indicated strains. Mature leaves of 5-week-old plants were infiltrated, and a representative leaf was photographed 36 h after infiltration (hai). Red dotted lines indicate infiltrated regions. **(B)** Ion conductivity in leaves infiltrated with the indicated strains. At 0 and 24 hai, leaf disks were excised to measure electrolyte leakage. Error bars indicate SD (*n* = 4). Different letters indicate statistically significant differences as determined by Duncan’s multiple range test (*p* < 0.05). Mock, 10 mM MgCl_2_; WT, *C. michiganensis* LMG7333 wild type.

Previously, it was shown that *Clavibacter* species induced HR in nonhost plant species, and that the *chp-7_Cs_* gene, a homolog of *pat-1_Cm_* that encodes a putative serine protease in *C. sepedonicus*, is involved in HR induction in *N. tabacum* (Nissinen et al., 2009). To determine if Pat-1*_Cm_* plays a role in *C. michiganensis* LMG7333 HR induction in a nonhost plant, we performed the HR assay in *N. tabacum* (cv. *Samsun*) plants. Infiltration of WT *C. michiganensis* into *N. tabacum* leaves induced a typical HR within 24 h after infiltration (hai); however, an HR was not induced by the mutant strains, Tn::*pat-1_Cm_* (Figure 3A) and 7333ΔpCM2 (Supplementary Figure 6). Moreover, the HR-eliciting ability of both mutants was restored by complementation with an intact *pat-1_Cm_* gene. To quantify *C. michiganensis*-triggered HR, we measured electrolyte leakage during HR induction in tobacco leaves. Compared to the WT, electrolyte leakage after infiltration with the Tn::*pat-1_Cm_* strain was significantly and consistently reduced. Furthermore, complementing with the intact *pat-1_Cm_* restored electrolyte leakage in tobacco leaves to normal levels (Figure 3B). Our finding that the expression of *pat-1_Cm_* is directly associated with HR development in a nonhost plant suggests that the *pat-1_Cm_* gene in *C. michiganensis* is required not only for wilting development in a host plant, but also for HR induction in a nonhost plant.

### The *chp-7_Cs_* Is Functionally Conserved With *pat-1_Cm_* in Pathogenicity

The *pat-1_Cm_* orthologs were found in several species, including *C. michiganensis*, *C. capsici*, and *C. sepedonicus*. Those genes, *pat-1_Cc_*, *pat-1_Cs_*, and *chp-7_Cs_*, are conserved in their plasmids with amino acid sequences that are approximately 77% identical to *pat-1_Cm_* (Supplementary Figure 4A). The *chp-7_Cs_*, which is a chromosomal putative serine protease of *C. sepedonicus*, was also classified as a *pat-1_Cm_* ortholog. A functional SP was predicted in Pat-1_Cc_ and Chp-7_Cs_, but not in Pat-1_Cs_ (Supplementary Figure 4A).

**Figure 4:**
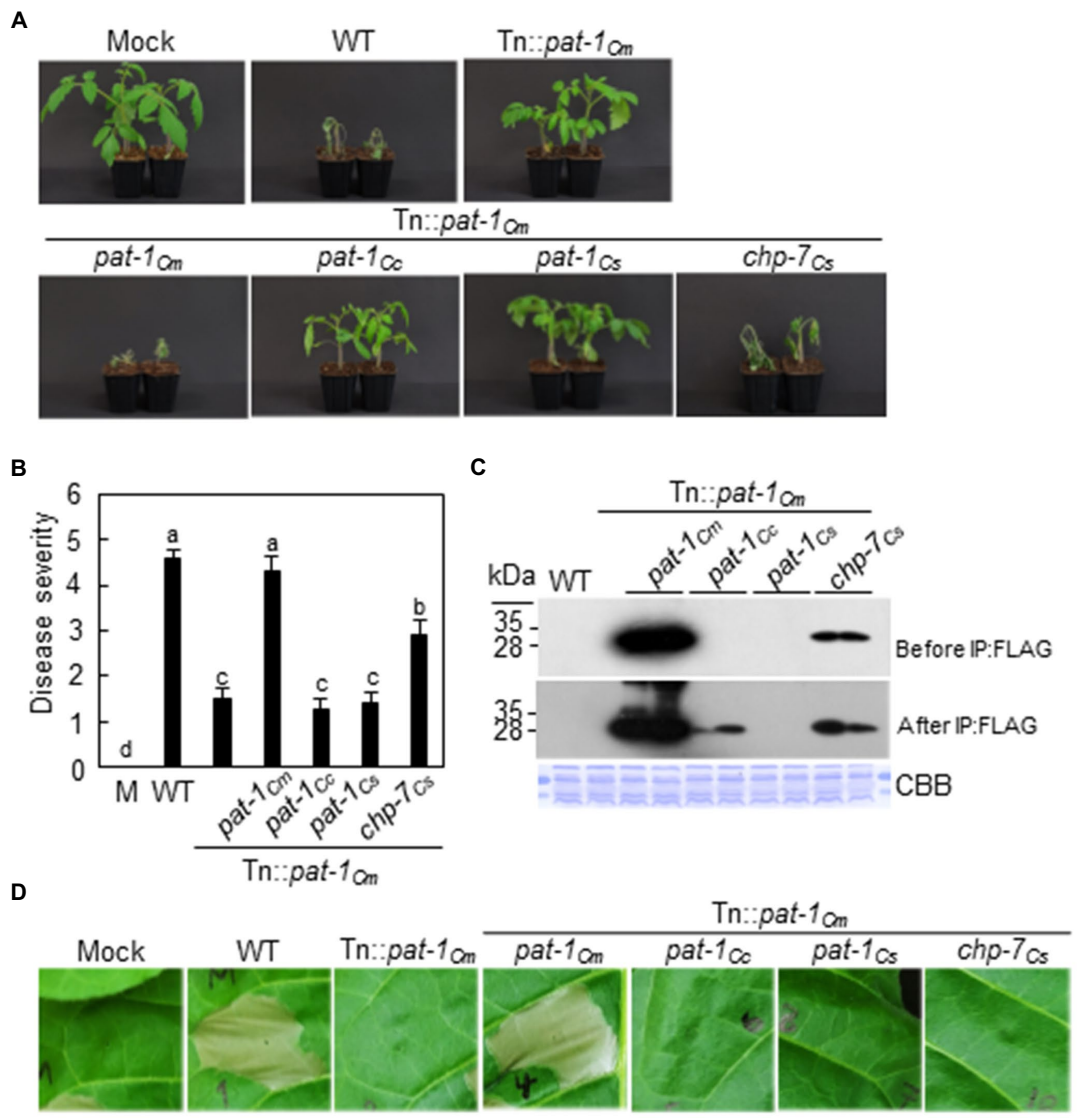
Partial recovery of *Clavibacter michiganensis* LMG7333 Tn::*pat-1_Cm_* mutant virulence by *chp-7Cs* gene of *C. sepedonicus*. **(A)** Wilting symptom development by inoculation of Tn::*pat-1_Cm_* strain carrying the *pat-1_Cm_* and its orthologs. Inoculated plants were observed for 2 weeks and photographed 14 dai. **(B)** Disease severity of wilting in tomato plants inoculated with the indicated strains at 14 dai. Error bars indicate SE (*n* = 10). Nonparametric Kruskal–Wallis test with Dunnett’s multiple comparisons (*p* < 0.05) was used to analyze the level of disease severity in tomato plants, and different letters indicate statistically significant differences at *p* < 0.05. **(C)** Expression and secretion of Pat-1_Cm_ and its orthologs in the complemented strains. All proteins were fused to FLAG on their C-termini and expressed in Tn::*pat-1_Cm_*. Total proteins from supernatant before and after immunoprecipitation (IP:FLAG) with anti-FLAG-agarose beads were analyzed by immunoblot using anti-FLAG antibody. CBB, Coomassie Brilliant Blue. **(D)** HR phenotype in *Nicotiana tabacum* after infiltration with the indicated strains. Leaves of 5-week-old plants were inoculated, and representative leaves were photographed 36 h after infiltration. Red dotted lines indicate infiltrated regions. Mock, 10 mM MgCl_2_.

Subsequently, we determined if the *pat-1_Cm_* orthologs confer virulence of by complementing *C. michiganensis* Tn::*pat-1_Cm_* with each of *pat-1_Cc_*, *pat-1_Cs_*, and *chp-7_Cs_* genes under control of a 0.4-kb native promoter of *pat-1_Cm_* (Supplementary Figure 4B). The Tn::*pat-1_Cm_* strain with *chp-7_Cs_* caused wilting in tomato, but disease severity was significantly less than with the WT *C. michiganensis* (Figures 4A,B). The amount of expressed and secreted Chp-7_Cs_ proteins in Tn::*pat-1_Cm_* was significantly lower than that of Pat-1_Cm_ (Figure 4C). In contrast, the strains with either *pat-1_Cc_* or *pat-1_Cs_* failed to recover virulence activity (Figures 4A,B). When their expression and secretion levels were examined, Pat-1_Cc_ was expressed and secreted, but less than Chp-7_Cs_, and was only detected after immunoprecipitation with FLAG affinity beads (Figure 4C). As expected, due to the lack of the N-terminal SP on Pat-1_Cs_, it was not detected. Next, the HR induction was examined with complemented strains. Complementation with *pat-1_Cm_* induced a strong HR similar to the WT, but none of the other three complemented strains could induce HR in *N. tabacum* leaves (Figure 4D). These results indicate that the virulence of only *chp-7_Cs_* is conserved with *pat-1_Cm_* and that the amount of secreted proteins might be directly correlated with disease severity.

### Roles of Pat-1*_Cm_* in Pathogenicity and HR Elicitation Are Distinct From Other Chp proteins

**Figure 5:**
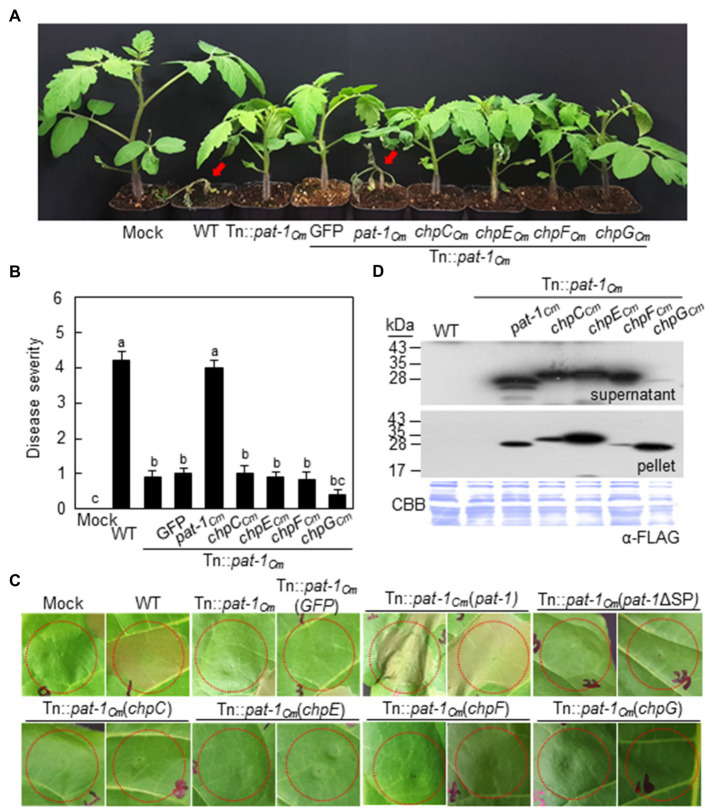
No recovery of either pathogenicity or HR-eliciting activity of *Clavibacter michiganensis* mutant Tn::*pat-1_Cm_* by chromosomal homologs of *pat-1_Cm_* (*chp*) genes. **(A)** Wilting symptom development after inoculation with Tn::*pat-1_Cm_* carrying *chpC_Cm_*, *chpE_Cm_*, *chpF_Cm_*, or *chpG_Cm_*. Inoculated plants were observed for 2 weeks and photographed 14 dai. **(B)** Disease severity of wilting in tomato plants inoculated with the indicated strains at 14 dai. Error bars indicate SE (*n* = 10). Nonparametric Kruskal–Wallis test with Dunnett’s multiple comparisons (*p* < 0.05) was used to analyze the level of disease severity in tomato plants, and different letters indicate statistically significant differences at *p* < 0.05. **(C)** HR phenotype in *Nicotiana tabacum* after infiltration with the indicated strains. Leaves of 5-week-old plants were inoculated, and representative leaves were photographed at 36 h after infiltration. Red dotted lines indicate the infiltrated regions. **(D)** Expression and secretion of Pat-1_Cm_ and Chp proteins in complemented strains. All proteins were fused to FLAG on their C-termini and expressed in Tn::*pat-1_Cm_*. Total proteins from the supernatant and pellet were analyzed by immunoblot using anti-FLAG antibody. CBB, Coomassie Brilliant Blue. Mock, 10 mM MgCl_2_; WT, *C. michiganensis* LMG7333 wild type.

In the chromosome of *C. michiganensis* strain LMG7333, there are seven genes (*chpA_Cm_* to *chpG_Cm_*) known as chromosomal homology to *pat-1* (*chp*; Stork et al., 2008). Three (*chpA_Cm_*, *chpB_Cm_*, and *chpD_Cm_*) are pseudogenes containing internal stop codons, whereas the remaining four genes encode mature forms of putative serine proteases. These latter four proteins have a putative SP sequence and were functionally classified as a serine protease family. However, protein sequence identities between these four proteins and Pat-1*_Cm_* were lower than 40% (Supplementary Figures 7A,B). To determine if any of these *chp* genes could functionally complement *pat-1_Cm_*, full length (FL) *chpC_Cm_*, *chpE_Cm_*, *chpG_Cm_*, and *chpF_Cm_* genes tagged with FLAG on their C-termini were transformed into both Tn::*pat-1_Cm_* and 7333ΔpCM2, and then strains’ ability to cause wilting in tomato and to elicit HR in *N. tabacum* was examined. Intriguingly, none of the four *chp* genes enabled either mutant to cause wilting in tomato (Figures 5A,B; Supplementary Figures 8A,B) or to elicit HR in *N. tabacum* (Figure 5C; Supplementary Figure 8C), even though they were expressed and secreted similar to Pat-1_Cm_ proteins (Figure 5D). These results indicate that the manner in which Pat-1_Cm_ interacts with plants is distinct from other homologous Chp proteases.

### The Conserved Catalytic Triad for Putative Serine Protease in Pat-1_Cm_ Is Critical for Disease Development and HR Induction in Plants

**Figure 6:**
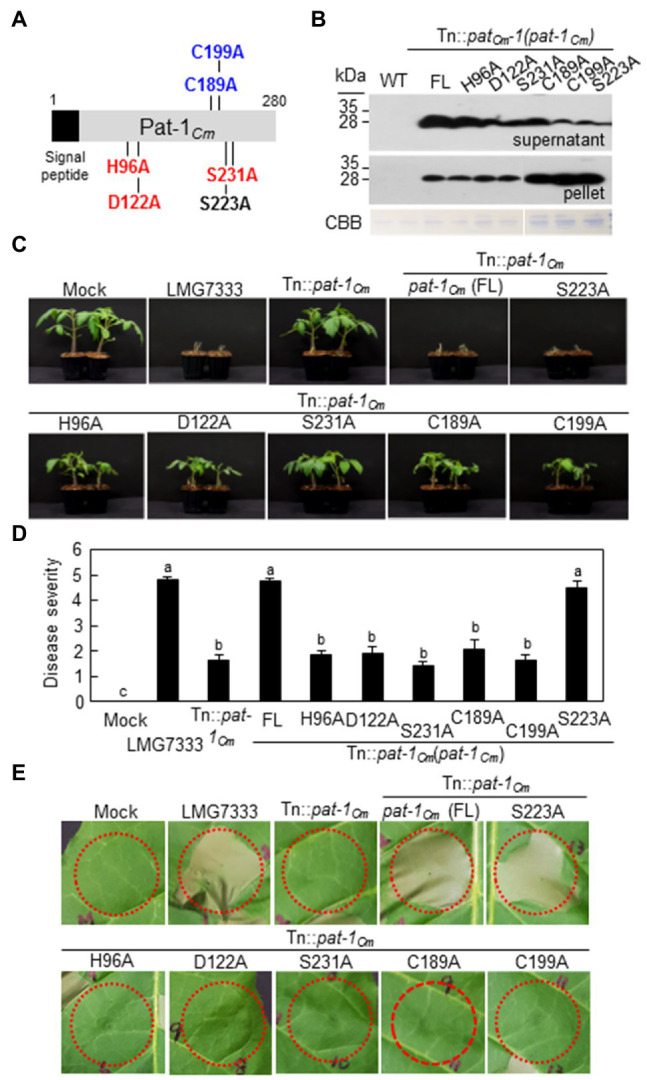
Key amino acids in the Pat-1_Cm_ protein for both pathogenicity and HR-eliciting activities of *Clavibacter michiganensis*. **(A)** Schematic of amino acid substitutions in the Pat-1_Cm_ protein. Variants with catalytic triad replacements (H96A, D122A, and S231A) and disulfide bond replacements (C189A and C199A) are shown in red and blue, respectively. Variant S222A was randomly selected as a control. **(B)** Expression and secretion of Pat-1_Cm_ and its variants in the indicated strains. All proteins were fused to FLAG on their C-termini and expressed in Tn::*pat-1_Cm_* strains. Total proteins from supernatant and pellet were analyzed by immunoblot using anti-FLAG antibody. CBB, Coomassie Brilliant Blue. **(C)** Wilting symptom development in tomato plants inoculated with *C. michiganensis* Tn::*pat-1_Cm_* strain carrying diverse variants of the full length (FL) *pat-1_Cm_* gene. Two-week-old plants were inoculated and were photographed 14 dai. **(D)** Disease severity of wilting in tomato plants inoculated with the indicated strains 14 dai. Error bars indicate SE (*n* = 10). Nonparametric Kruskal–Wallis test with Dunnett’s multiple comparisons (*p* < 0.05) was used to analyze the level of disease severity in tomato plants, and different letters indicate statistically significant differences at *p* < 0.05. **(E)** HR phenotype in *Nicotiana tabacum* after infiltration with indicated strains. Five-week-old plant leaves were inoculated, and representative leaves were photographed 36 h after infiltration. Red dotted lines indicate infiltrated regions. Mock, 10 mM MgCl_2_; FL, full length *pat-1_Cm._*

Pat-1*_Cm_* is considered a putative serine protease due to its C-terminal conserved motif, Gly-ASP-Ser-Gly-Gly (GDSGG; Krem et al., 1999; Burger et al., 2005; Nissinen et al., 2009). Here, Pat-1*_Cm_* protein structures were predicted using the Phyre2 program to determine the key functional amino acid residues. Protein modeling showed that Pat-1*_Cm_* protein contains three residues in the following order histidine (His, H96), aspartate (Asp, D122), and serine (Ser, S231). This sequence is consistent with the catalytic triad of well-characterized serine proteases (Figure 6A; Supplementary Figure 9). The Ser/His/Asp catalytic triad is highly conserved in all Pat-1 proteins from *Clavibacter* species (Supplementary Figure 4A) and the Chp protein family in the chromosome of LMG7333 (Supplementary Figure 7A). In addition, protein modeling showed that the Pat-1*_Cm_* protein has two cysteine residues at positions 189 and 199 that are connected by disulfide bond to form a proper tertiary structure (Supplementary Figure 9). Similar to the catalytic triad, two cysteine residues are found in Pat-1 proteins and the Chp protein family except for ChpC (Supplementary Figures 4A, 7A).

To investigate if the five residues in the catalytic triad (H96, D122, and S231) and the disulfide bond (C189, C199) play crucial roles in the function of Pat-1_Cm_, the individual residues were substituted with alanine by site-directed mutagenesis (Figure 6A) and introduced into the mutant strain, Tn:: *pat-1_Cm_*. Additionally, the serine residue at position 223 (Ser, S223) located in the un-conserved region was substituted with alanine as an internal control to rule out the possibility that alanine substitution itself affects the protein structure. All alanine substituted proteins were expressed and secreted similar to the WT Pat-1_Cm_, and the S223A substituent was expressed the least (Figure 6B). The Tn:: *pat-1_Cm_* mutant strain carrying all alanine substitutions except for at S223A failed to restore the mutant’s abilities to cause wilting in tomato (Figures 6C,D) or to elicit HR in *N. tabacum* (Figure 6E). These results indicate that the catalytic triad for putative serine protease and the specific disulfide bond for proper tertiary structure formation is essential for Pat-1_Cm_ functioning in plants.

### Ectopic Expression of Secreted Pat-1*_Cm_* Proteins Alone Elicits HR in *Nicotiana tabacum* Leaves

**Figure 7:**
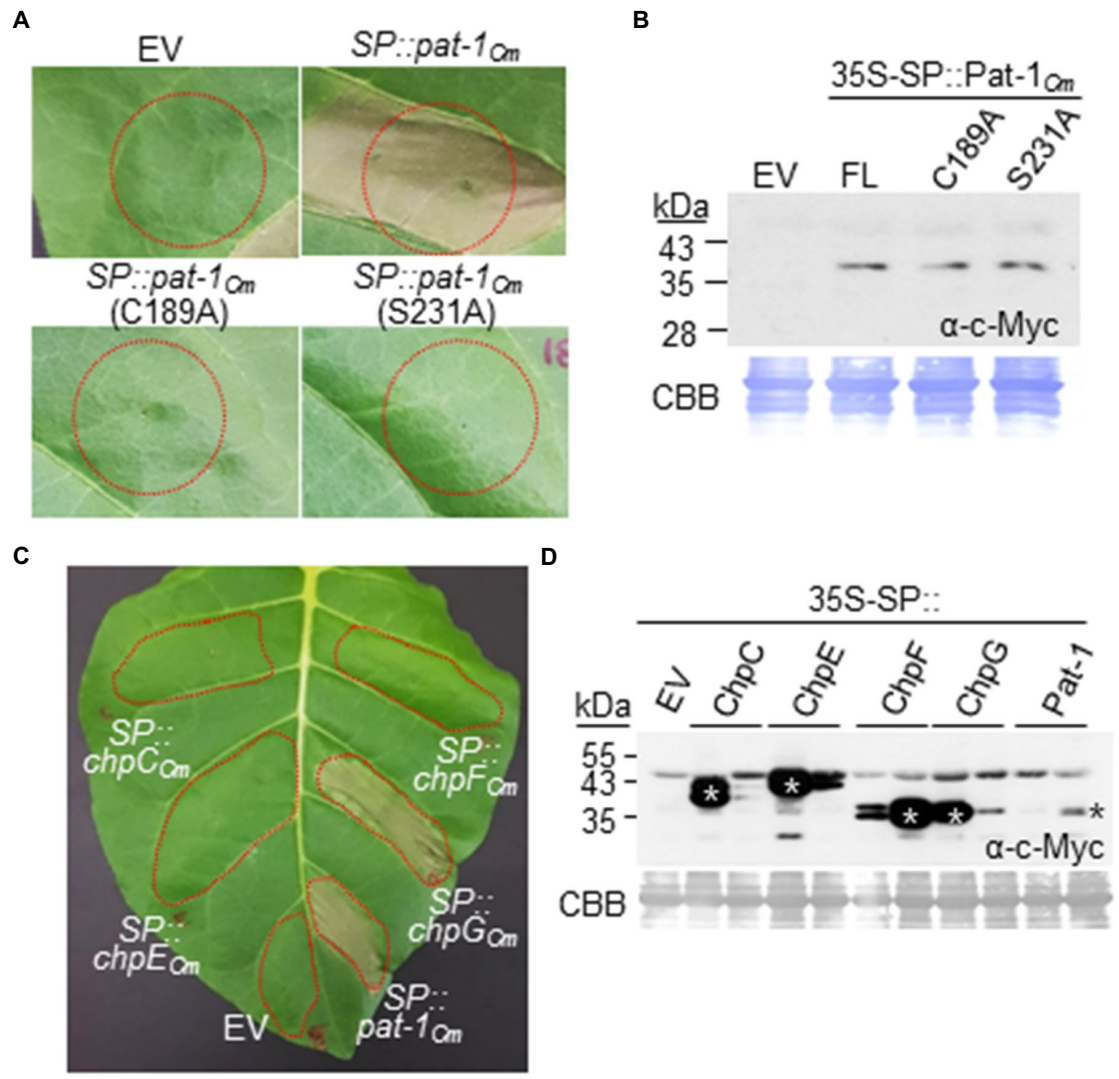
Hypersensitive response-eliciting activity of Pat-1_Cm_, its variants, and homologous Chp proteins by *Agrobacterium*-mediated transient expression in *Nicotiana tabacum*. Five-week-old *N. tabacum* leaves were infiltrated with *Agrobacterium* carrying each construct for constitutive expression of the indicated proteins with the SP of the tobacco PR1a protein. HR phenotypes in *N. tabacum* by expression of Pat-1_Cm_ and its variants **(A)** and homologous Chp proteins **(C)** were shown. Representative leaf was photographed 36 h after infiltration (hai). Red dotted lines indicate infiltrated regions. Empty vector (EV) was used as a negative control. **(B,D)** Western blotting of transiently expressed proteins tagged with c-Myc in their C-termini using c-Myc antibody. Infiltrated leaves were sampled at the time point before HR was fully progressed, and total proteins were extracted. * in **(D)** indicates the right bands for each protein. CBB, Coomassie Brilliant Blue.

We showed that the mutant Tn::*pat-1_Cm_* lost its ability to elicit HR in a nonhost plant (Figure 3). To determine whether the Pat-1_Cm_ protein alone elicits HR, the SP of tobacco PR1a was fused to the N-terminus of full-length Pat-1*_Cm_* to mimic Pat-1_Cm_ secretion and translocation to the apoplast of *N. tabacum* leaves. In addition, a c-Myc tag was fused to the C-terminus of Pat-1_Cm_ to check protein expression. The final construct, 35S-SP::Pat-1_Cm_ under control of the 35S promoter, was transformed into *Agrobacterium* bacterium. *Agrobacterium*-mediated transient expression of Pat-1*_Cm_* in *N. tabacum* leaves, but not in a host plant tomato, elicited HR within 48 h, whereas the empty vector (EV) did not (Figure 7A; Supplementary Figure 10). Since the catalytic triad and disulfide bond are important for Pat-1*_Cm_* functioning, we generated two more constructs carrying Pat-1*_Cm_*–C189A and Pat-1*_Cm_*–S231A and expressed in *N. tabacum* leaves. These two variants failed to elicit HR, unlike the WT Pat-1*_Cm_* (Figure 7A), although they were expressed at the same level as the WT Pat-1*_Cm_* (Figure 7B). These results indicate that the Pat-1_Cm_ protein alone, when located in plant apoplast, can elicit HR in a nonhost plant. In addition, the results suggest that both the catalytic triad for putative serine protease and a disulfide bond dependent structure in Pat-1_Cm_ are required for HR induction in a nonhost plant.

Although four intact Chp proteins in *C. michiganensis*, ChpC*_Cm_*, ChpE*_Cm_*, ChpF*_Cm_*, and ChpG*_Cm_*, could not complement the Tn::*pat-1_Cm_* mutant to restore its HR-eliciting ability (Figure 5C), we checked whether any of these four Chp proteins alone could elicit HR when ectopically expressed on *N. tabacum* leaves. An HR was induced by expression of ChpG*_Cm_*, but not ChpC*_Cm_*, ChpE*_Cm_*, or ChpF*_Cm_* (Figure 7C). All four proteins were expressed at higher levels than Pat-1_Cm_ (Figure 7D). These results indicate that, although ChpG*_Cm_* protein alone can elicit HR, it cannot substitute the ability of Pat-1_Cm_ in *C. michiganensis* to elicit HR in *N. tabacum*.

### Pat-1_Cm_ Can Cooperate With CelA, a Secreted Cellulase, to Cause Wilting in Tomato

**Figure 8:**
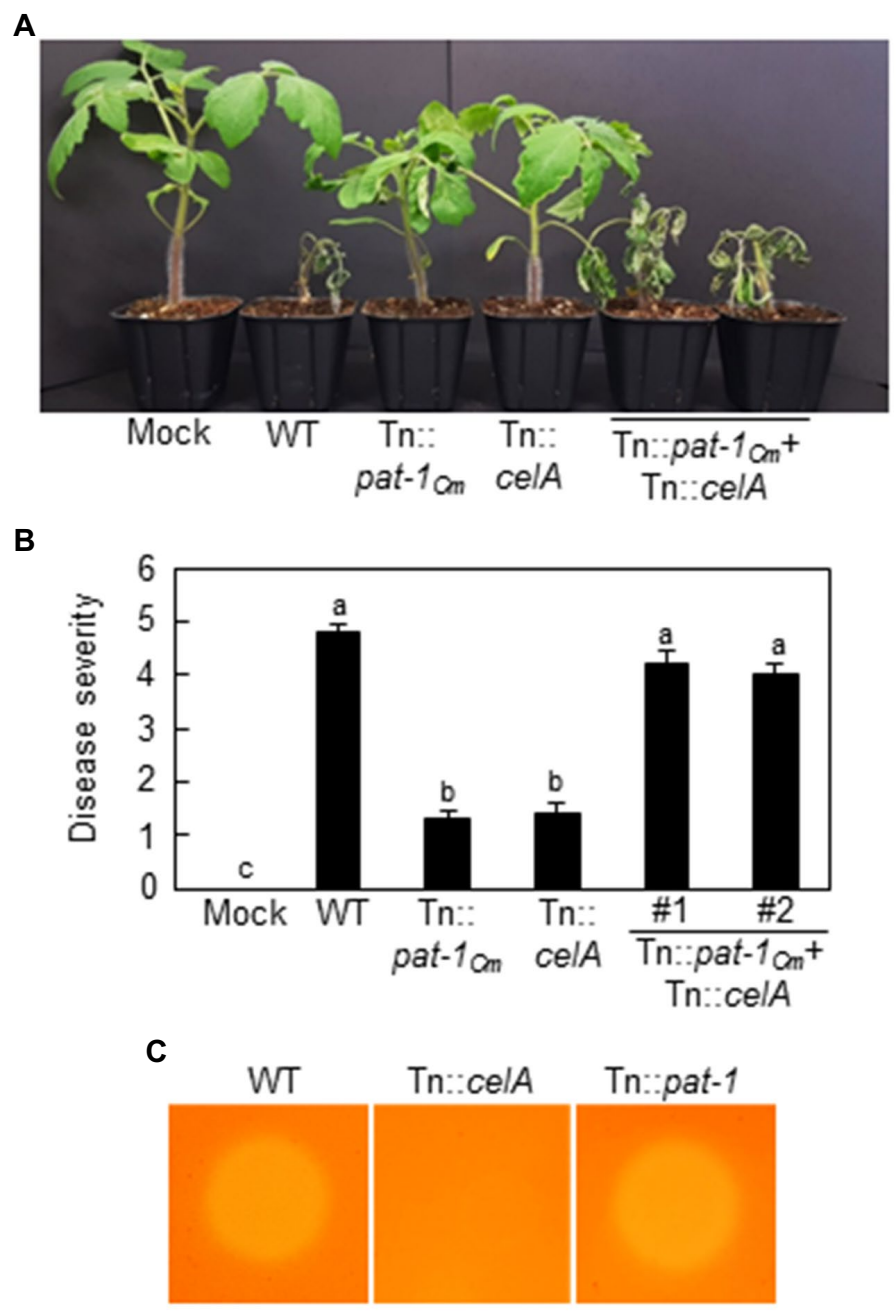
Functional cooperation of Pat-1_Cm_ with another major pathogenicity factor, CelA (cellulase), during *Clavibacter michiganensis* infection of tomato plants. **(A)** Wilting symptom development in tomato plants inoculated with individual or mixed strains. Two-week-old plants were inoculated, and disease symptoms were photographed 14 dai. **(B)** Disease severity of wilting in tomato plants inoculated with mixed strains at 14 dai. Error bars indicate SE (*n* = 10). Nonparametric Kruskal–Wallis test with Dunnett’s multiple comparisons (*p* < 0.05) was used to analyze the level of disease severity in tomato plants, and different letters indicate statistically significant differences at *p* < 0.05. **(C)** Plate assay for detection of cellulase activity in Tn::*celA* and Tn::*pat-1_Cm_* mutant strains using carboxymethyl cellulose (CMC) agar plates strained with Congo Red. Mock, 10 mM MgCl_2_; WT, *C. michiganensis* LMG7333 wild type.

Previously, we reported that *celA*, another plasmid-borne pathogenicity gene in *C. michiganensis* that encodes a secreted cellulase, is critical for the development of wilting in tomato (Hwang et al., 2019). Because both Pat-1_Cm_ and CelA encode secreted proteins with different enzymatic activities, we hypothesized that both proteins originated from different mutant bacteria present in the same site can functionally cooperate to cause wilting in tomato. To test this hypothesis, mutants Tn::*celA* and Tn::*pat-1_Cm_* were inoculated individually or co-inoculated on 2-week-old tomato plants by the root-dipping inoculation method. The individually inoculated plants did not show wilting, whereas the co-inoculated plants showed severe wilting, similar to WT-inoculated plants (Figures 8A,B). Moreover, cellulase activity similar to that of the WT strain was observed in Tn::*pat-1_Cm_*, whereas Tn::*celA* lost its ability to produce cellulase (Figure 8C). These results indicate that two different but important pathogenicity proteins, CelA and Pat-1*_Cm_*, function cooperatively to cause wilting in a host plant.

## Discussion

In this study, we determined that *C. michiganensis* Pat-1_Cm_ is a genuine secreted protein with a functional SP and acts as both a pathogenicity factor in a host plant tomato and an HR inducer in a nonhost plant *N. tabacum*. To our knowledge, Pat-1_Cm_ and its close ortholog, Chp-7_Cs_, are only proteins to perform both activities in Gram-positive plant pathogenic bacteria, similar to the Hrp proteins in Gram-negative bacteria. Hrp proteins function mostly as structural proteins to form T3SS in a bacterial plasma membrane, whereas Pat-1_Cm_ is secreted and very likely localizes to the apoplastic space to function. Based on genomic analyses, *C. michiganensis* does not have T3SS or T6SS, but probably has a T2SS and a TAT secretion system (Supplementary Table 2). Because the secreted mature form of Pat-1_Cm_ does not have an SP, suggesting that the SP is cleaved during secretion, this protein might be secreted by either T2SS or TAT secretion system, although this inference remains to be confirmed. The SP appears to be crucial for the correct expression and secretion of Pat-1_Cm_ because no Pat-1_Cm_ΔSP protein was detected in either the pellet or supernatant, when the protein was expressed in the Tn::*pat-1_Cm_* mutant, whereas the SP-cleaved form of mature Pat-1_Cm_ from the WT was well detected.

Pat-1_Cm_ was previously predicted to be a chymotypsin-like serine protease with a catalytic triad for enzymatic activity (Rawlings et al., 2018), although such activity had not been observed yet. We tried to detect the enzymatic activity of mature Pat-1_Cm_ after purification and mixing with casein, a well-known protease substrate to evaluate if casein can be cleaved by Pat-1_Cm_, but not by Pat-1_Cm_ (S231A). However, we failed to observe the cleavage of casein protein (data not shown). This might indicate that the target(s) of mature Pat-1_Cm_ is specific and present only in host plants. Nevertheless, in this study, we showed that the catalytic triad of Pat-1_Cm_ for predicted serine protease is required for the protein to act as both a pathogenicity factor and an immunity elicitor. This implies that there might be a different target(s) of Pat-1_Cm_ in host and nonhost plants, if so, these targets need to be identified and characterized to fully understand the underlying mechanisms of Pat-1_Cm_.

Close orthologs of *pat-1_Cm_* have been reported in at least two more *Clavibacter* species, *C. capsici* (*pat-1_Cc_*) and *C. sepedonicus* (*pat-1_Cs_* and *chp-7_Cs_*), and are located on a large plasmid or a pathogenicity island in a chromosome (Dreier et al., 1997; Burger et al., 2005; Holtsmark et al., 2008). In addition, many *chp* family genes including *chpC*, *chpE*, *chpG*, and *chpF* that are less homologous to *pat-1_Cm_* than its close orthologs have been found in *Clavibacter* species (Eichenlaub and Gartemann, 2011; Hwang et al., 2020). When any of these orthologs or homologs were expressed in the *C. michiganensis* Tn::*pat-1_Cm_* mutant under the *pat-1_Cm_* promoter, none of them, except *chp-7_Cs_*, successfully replaced *pat-1_Cm_*’s pathogenicity function. Although *C. michiganensis chpC_Cm_* has been shown to be involved in virulence (Stork et al., 2008), it failed to restore *pat-1_Cm_* pathogenicity. Based on SP prediction and protein modeling, all of those proteins have an intact SP and a catalytic triad, indicating that these orthologs and homologs are very likely secreted proteins and act as putative serine proteases similar to Pat-1_Cm_. Therefore, a lack of functional conservation among Pat-1_Cm_ orthologs and homologs indicates that each protein might have a different target(s) in plants during infection.

Interestingly, Chp-7_Cs_ and ChpG_Cm_, which have been shown to trigger HR in *N. tabacum* leaves (Lu et al., 2015), failed to replace the HR-eliciting activity of Pat-1_Cm_. Although the amount of secreted Chp-7_Cs_ proteins was significantly less than Pat-1_Cm_, it was sufficient for replacing Pat-1_Cm_ pathogenicity, but not for HR induction. The reason that the Tn::*pat-1_Cm_* mutant carrying the *chp-7_Cs_* gene did not induce HR might be related to the amount of Chp-7_Cs_ protein in the supernatant. Overall, Pat-1_Cm_ function in plants appears distinct from its close orthologs and homologs. Functional redundancy among serine proteases participating in pathogenicity or virulence in Gram-negative bacteria has been reported, whereas, in Gram-positive bacteria, it is limited (Xia, 2004; Figaj et al., 2019). Instead, these proteins might have evolved in concert with functional diversifications in *Clavibacter* species (Sacristan and Garcia-Arenal, 2008; Plissonneau et al., 2017). In the future, identifying the protein(s) targeted by Pat-1_Cm_ in host and nonhost plants will help to clarify the molecular mechanisms of Pat-1_Cm_ functioning beyond pathogenicity.

The Pat-1_Cm_ catalytic triad was necessary for pathogenicity and HR induction. Pat-1_Cm_ alanine substituents were stably expressed and secreted, indicating that the catalytic triad is not necessary for protein stability, but critical for function. This means that the protein’s activity as a serine protease might be critical for pathogenicity and HR induction. Protease families containing a catalytic triad in plant pathogenic bacteria appear structurally similar to those found in human and animal pathogens, implying that these proteases also might play a role in pathogenicity or virulence depending on the proteolytic activity mediated by these residues (Figaj et al., 2019). Additionally, disulfide bond formation between pairs of cysteine residues is involved in bacterial virulence by contributing to protein stability (Reardon-Robinson and Ton-That, 2015; Smith et al., 2016). Dsb (disulfide bond formation) family proteins known to catalyze disulfide bonding are found in many plant-pathogenic bacteria, such as *Xanthomonas campestris* pv. *campestris* and *Pseudomonas aeruginosa* (Braun et al., 2001; Jiang et al., 2008). Disulfide bonding in many bacterial-secreted proteins is directly involved in the protein structural stability by encouraging proper folding (Heras et al., 2009). Two cysteines (C189 and C199) were predicted to form a disulfide bond, and alanine substituents disabled Pat-1_Cm_ pathogenicity and HR induction. Because their protein expression and secretion appeared normal like the WT, this disulfide bond is likely important for maintaining this protein’s proper structure.

Co-inoculation of tomato with *C. michiganensis* Tn::*celA* and Tn::*pat-1_Cm_* mutants caused almost the same degree of wilting as observed after inoculation like the WT strain. *celA* encodes a secreted cellulase, which is important for wilting development in tomato (Hwang et al., 2019). After these two proteins are secreted during infection, it is highly likely that they move to the apoplast together and then function cooperatively regardless of their distinct origins. Thus far, the infection route of *C. michiganensis* is unknown. As shown previously (Hwang et al., 2019) and in this study, both *C. michiganensis* Tn::*celA* and Tn::*pat-1_Cm_* mutants possessed the ability to cause canker symptom, implying that these two genes are critical for wilting development. If this pathogen infects host plants through their roots, then it should pass several cell layers to reach xylem vessels for wilting development and systemic movement. Where CelA and Pat-1_Cm_ are needed during this process should be dissected in detail.

Overall, we provide a unique apoplastic effector of Gram-positive pathogenic bacterium with dual functions for interaction with host and nonhost plants, and the serine protease activity might be required for both interactions. This is an example of novel roles of serine proteases in plant-pathogen interactions. Moreover, the finding that the functional cooperation of two apoplastic effectors, a serine protease (Pat-1_Cm_) and a cellulase (CelA), is critical for pathogenicity in a host plant tells us a dynamic functional relationship among apoplastic effectors in plant-pathogen interactions.

## Data Availability Statement

The original contributions presented in the study are included in the article/Supplementary Material, further inquiries can be directed to the corresponding author.

## Author Contributions

IH, E-JO, ES, DC, and C-SO designed the study, performed the experiments, and drafted the manuscript. YL and KS performed the genome analysis. IH, E-JO, IP, YL, KS, DC, and C-SO substantially revised the manuscript. All authors contributed to the article and approved the submitted version.

## Funding

This work was supported by the National Research Foundation of Korea (NRF) grant funded by the Korean government (MSIT; 2019R1A2C2004568 and 2018R1A5A1023599, SRC).

## Conflict of Interest

The authors declare that the research was conducted in the absence of any commercial or financial relationships that could be construed as a potential conflict of interest.

## Publisher’s Note

All claims expressed in this article are solely those of the authors and do not necessarily represent those of their affiliated organizations, or those of the publisher, the editors and the reviewers. Any product that may be evaluated in this article, or claim that may be made by its manufacturer, is not guaranteed or endorsed by the publisher.

## Footnotes

^1^ http://www.sbg.bio.ic.ac.uk/~phyre2/html/page.cgi?id=index
