# Supplementary material for: An Apoplastic Effector Pat-1Cm of the Gram-Positive Bacterium Clavibacter michiganensis Acts as Both a Pathogenicity Factor and an Immunity Elicitor in Plants
Source: Front Plant Sci. 2022 Mar 30;13:888290. doi: 10.3389/fpls.2022.888290 (PMC9006514; doi:10.3389/fpls.2022.888290)
Supplement: Supplementary file 1 [file Presentation_1.pdf]

Supplementary Material

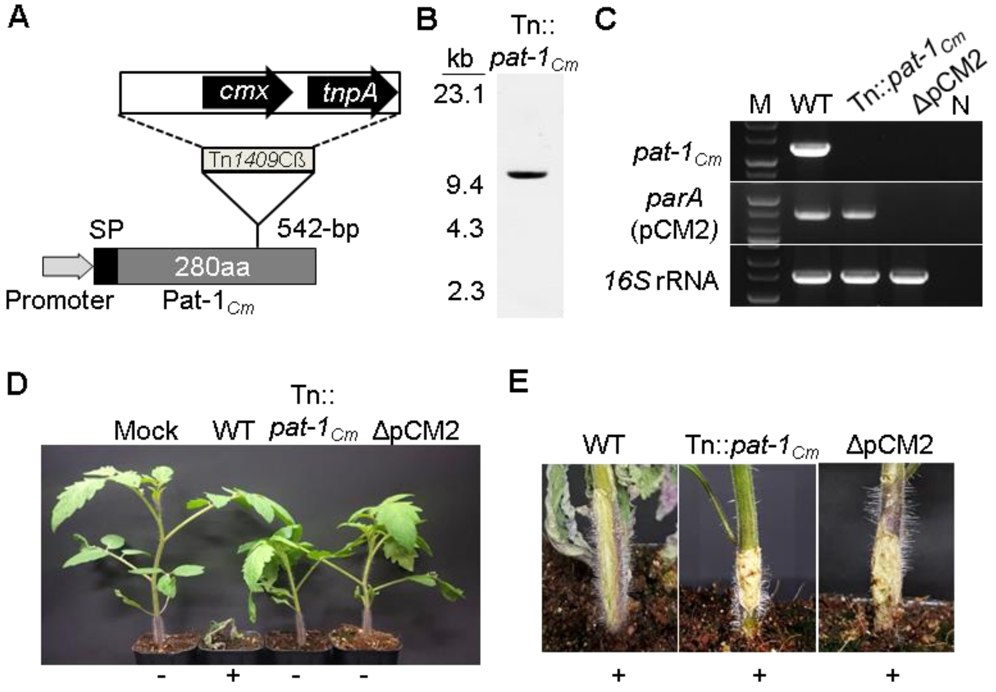

Supplementary Figure 1 Loss of pathogenicity of *Clavibacter michiganensis* in a host plant, tomato, by mutation of the *pat-1*$_{Cm}$ gene. **(A)** Schematic of Pat-1$_{Cm}$ protein and the 542-bp insertion site of transposon Tn*1409*Cβ carrying a chloramphenicol resistance gene, *cmx*, and the transposase gene, *tnpA*. SP, signal peptide. **(B)** Verification of transposon insertion in *pat-1*$_{Cm}$ by Southern hybridization. Genomic DNA from a mutant was digested with *Sph*I and probed with the *cmx* gene from Tn*1409*Cβ. **(C)** Confirmation of transposon insertion in the mutant Tn::*pat-1*$_{Cm}$ and pCM2 deletion mutant (ΔpCM2) by PCR using specific primer sets to amplify *pat-1*$_{Cm}$, pCM2, and members of the *Clavibacter* genus. WT, type strain LMG7333; M, DNA marker; N, no DNA. **(D)** Wilting symptom of tomato plants inoculated with indicated strains. Two-week-old plants were used for this experiment, and disease symptoms were photographed at 14 days after inoculation (dai). **(E)** Bacterial canker on the stems of infected plants inoculated with indicated strains. These symptoms were photographed 14 dai. Mock, 10 mM $MgCl_2$; WT, *C. michiganensis* LMG7333 wild type. +, disease symptom; -, no disease symptom.

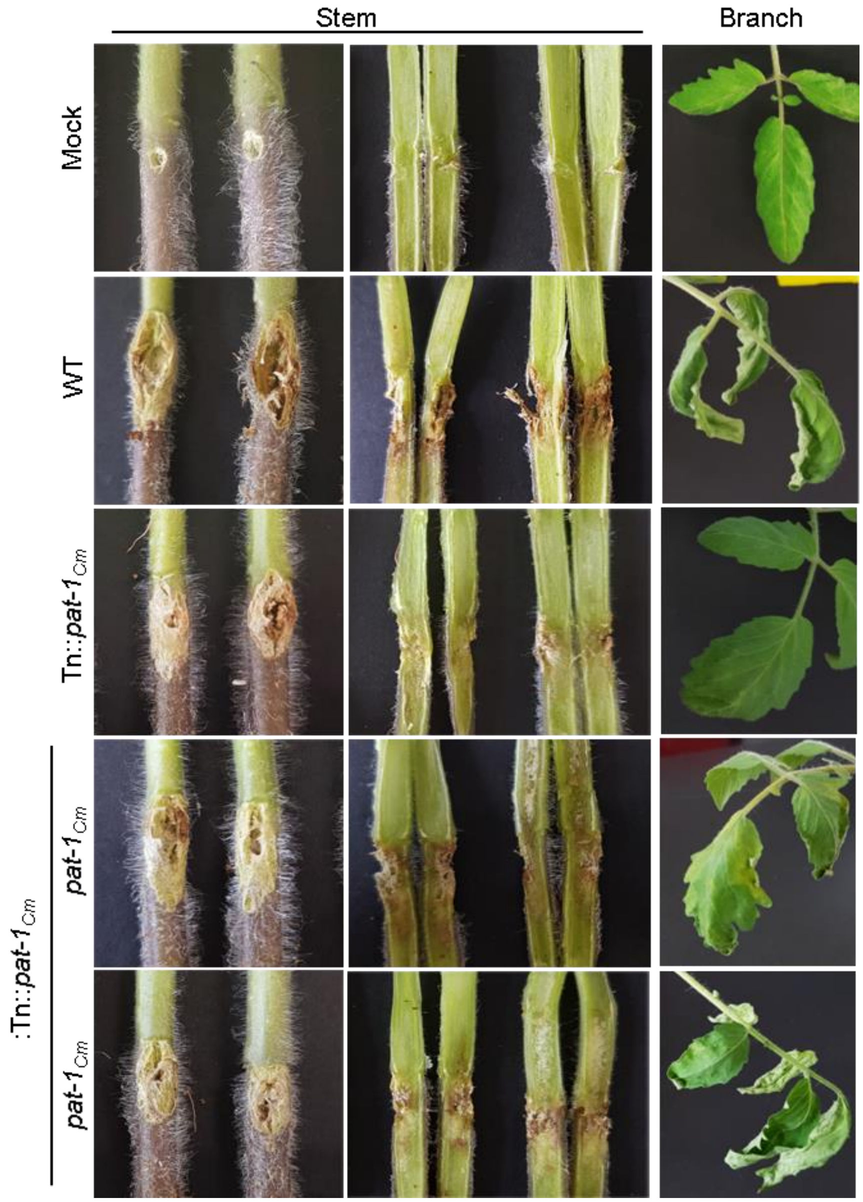

Supplementary Figure 2 Bacterial canker symptom on tomato stems after stem inoculation with *C. michiganensis* LMG7333 wild type (WT) and *pat-1*$_{Cm}$ mutant strains. Three-week-old plants were used for stem inoculation. External stem canker symptom, internal necrosis, and wilting symptom in the whole plant after stem inoculation were observed, and the representative photos were taken at 3 weeks after inoculation.

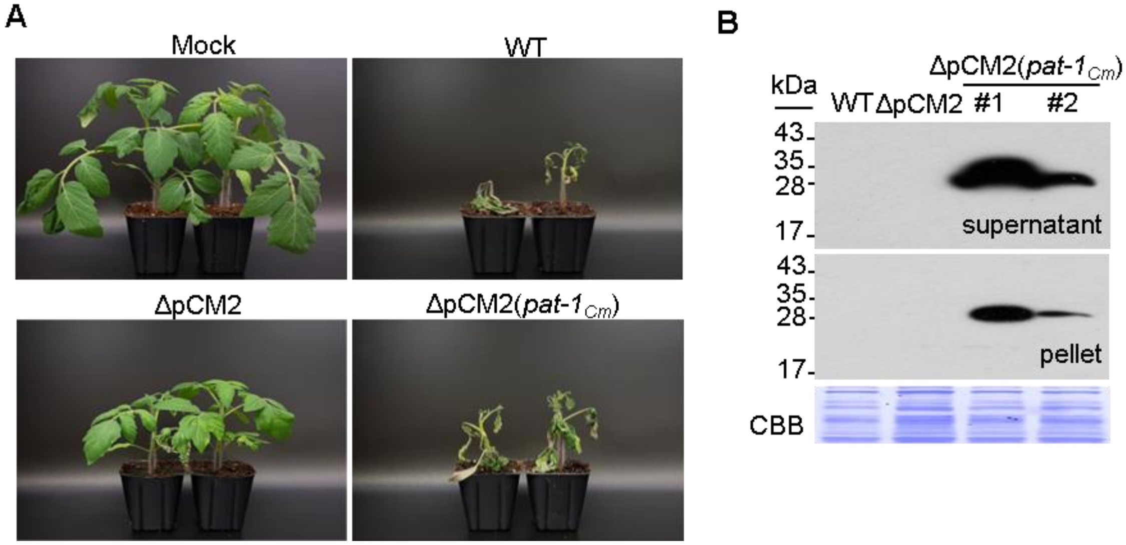

Supplementary Figure 3 Pathogenicity recovery of *C. michiganensis* ΔpCM2 mutant transformed with only the intact *pat-1*$_{Cm}$ gene in tomato. **(A)** Wilting symptom development in the infected tomato plants with the indicated strains. Complemented strains were generated by overexpressing full-length *pat-1*$_{Cm}$. Two-week-old plants were inoculated, and disease symptoms were photographed 14 days after inoculation (dai). **(B)** Expression and secretion of Pat-1$_{Cm}$ proteins in the complemented strains by western blotting. Pat-1$_{Cm}$ protein and its derivative were fused to FLAG on their C-termini and expressed in Tn::*pat-1*$_{Cm}$ strain. Total proteins from the supernatant and pellet were analyzed by immunoblotting using the FLAG antibody. CBB, Coomassie Brilliant Blue Mock, 10 mM $MgCl_2$; WT, *C. michiganensis* LMG7333 wild type.

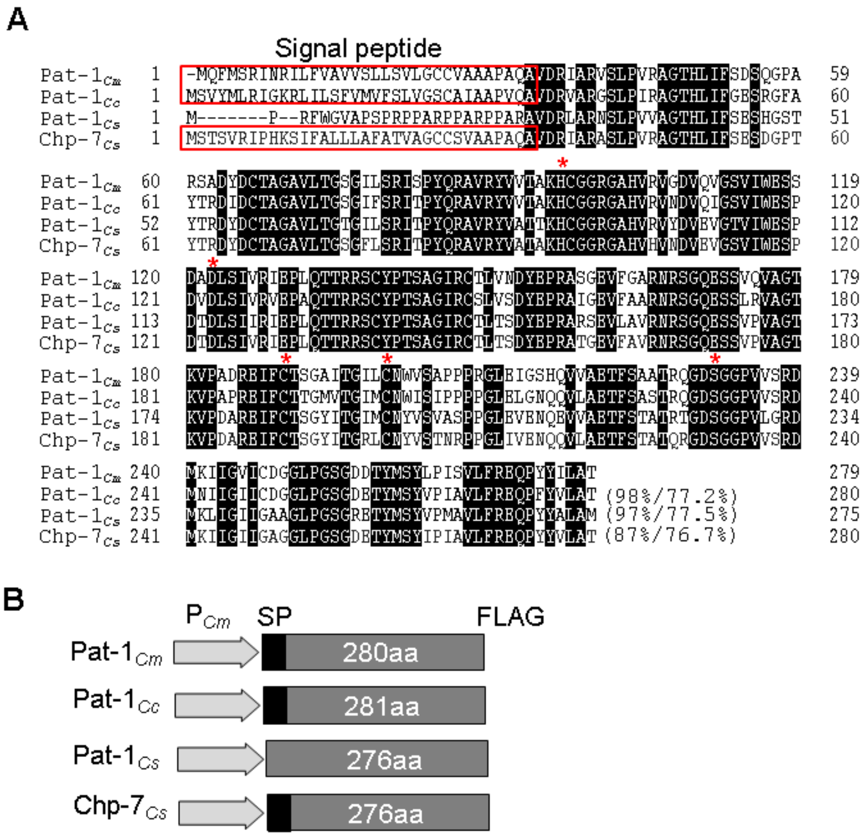

Supplementary Figure 4 Amino acid sequence of Pat-1 protein of *C. michiganensis* LMG7333. **(A)** Alignment of Pat-1 and its orthologs in other *Clavibacter* species, i.e., *C. capsici* (Cc) strain PF008 and *C. sepedonicus* (Cs) strain ATCC33113. Red box indicates putative signal peptide predicted with SignalP. * indicates amino acids for a catalytic triad and two key cysteines shown in Figure 6. Amino acid sequences were aligned using Clustal Omega. **(B)** Gene constructs for expression of Pat-1_{Cm} and its orthologs from other *Clavibacter* species. P_{cm}, pat-1_{Cm} native promoter; SP, signal peptide.

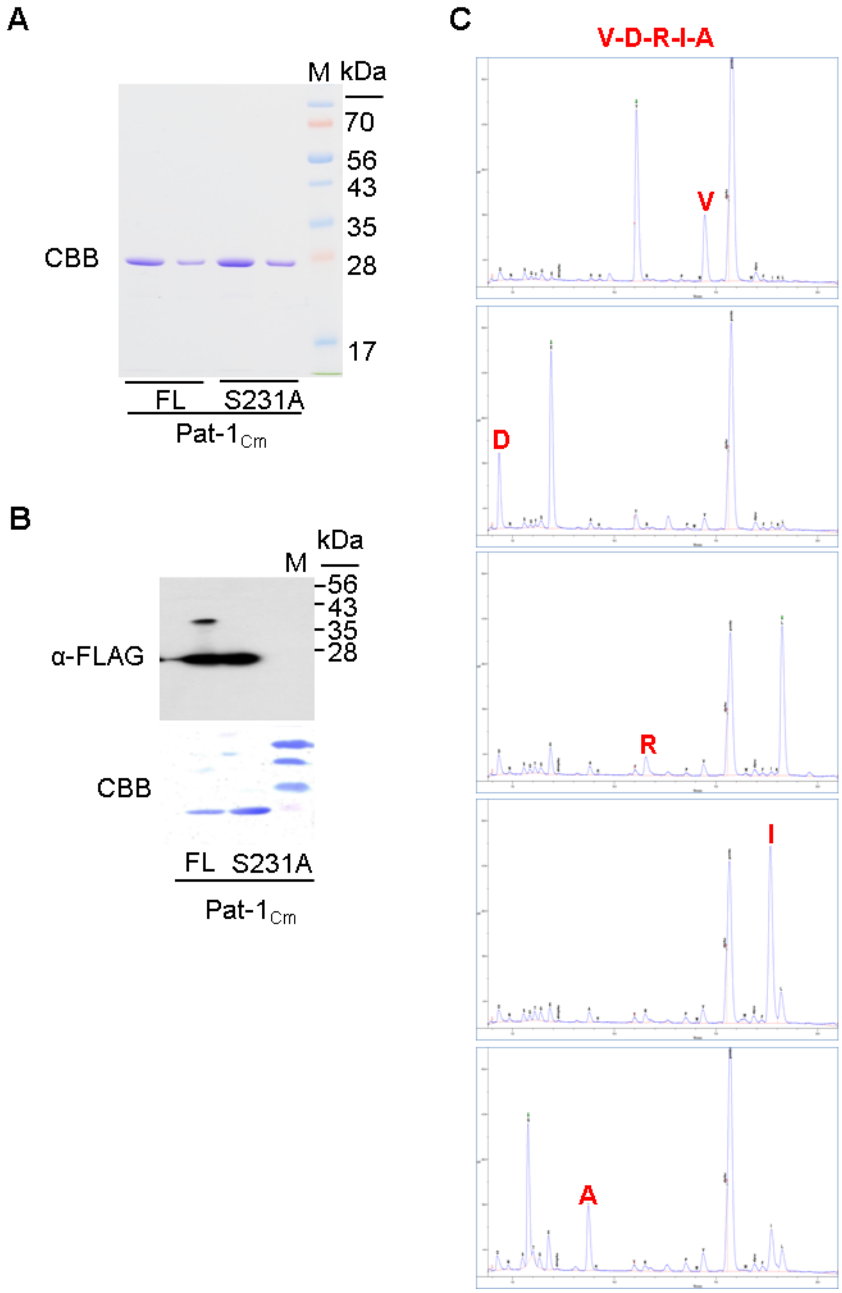

Supplementary Figure 5 N-terminal sequencing of mature Pat-1_{Cm} and Pat-1_{Cm}(S231A) proteins. **(A)** Collection of secreted mature protein from culture supernatant. **(B)** Western blot of mature Pat-1_{Cm} and Pat-1_{Cm}(S231A) proteins. (C) Identification of five N-terminal peptides in Pat-1_{Cm} by Edman degradation method shown in Figure 3.

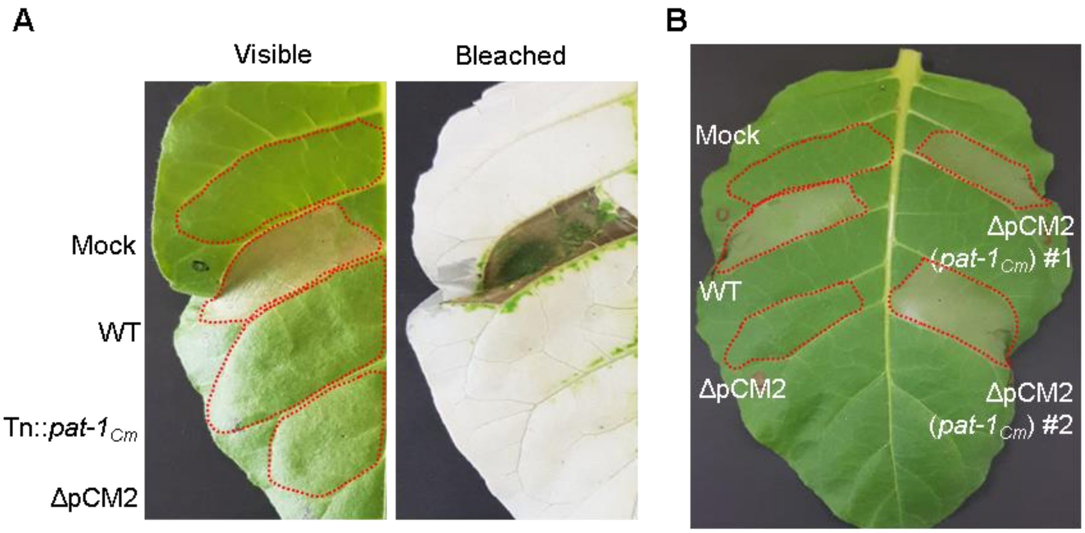

Supplementary Figure 6 Loss of HR-eliciting activity of *Clavibacter michiganensis* ΔpCM2 strain in a nonhost plant, *Nicotiana tabacum*. **(A)** HR phenotype in *N. tabacum* after infiltration with the indicated strains. Mature leaves of 5-week-old plants were infiltrated, and the representative leaf was photographed 36 h after infiltration (hai). The right panel shows the same leaf after ethanol bleaching for 24 h. Red dotted lines indicate the infiltrated regions. **(B)** HR recovery of ΔpCM2 strain by introduction of *pat-1*$_{Cm}$. Mock, 10 mM $MgCl_2$; WT, *C. michiganensis* LMG7333 wild type.

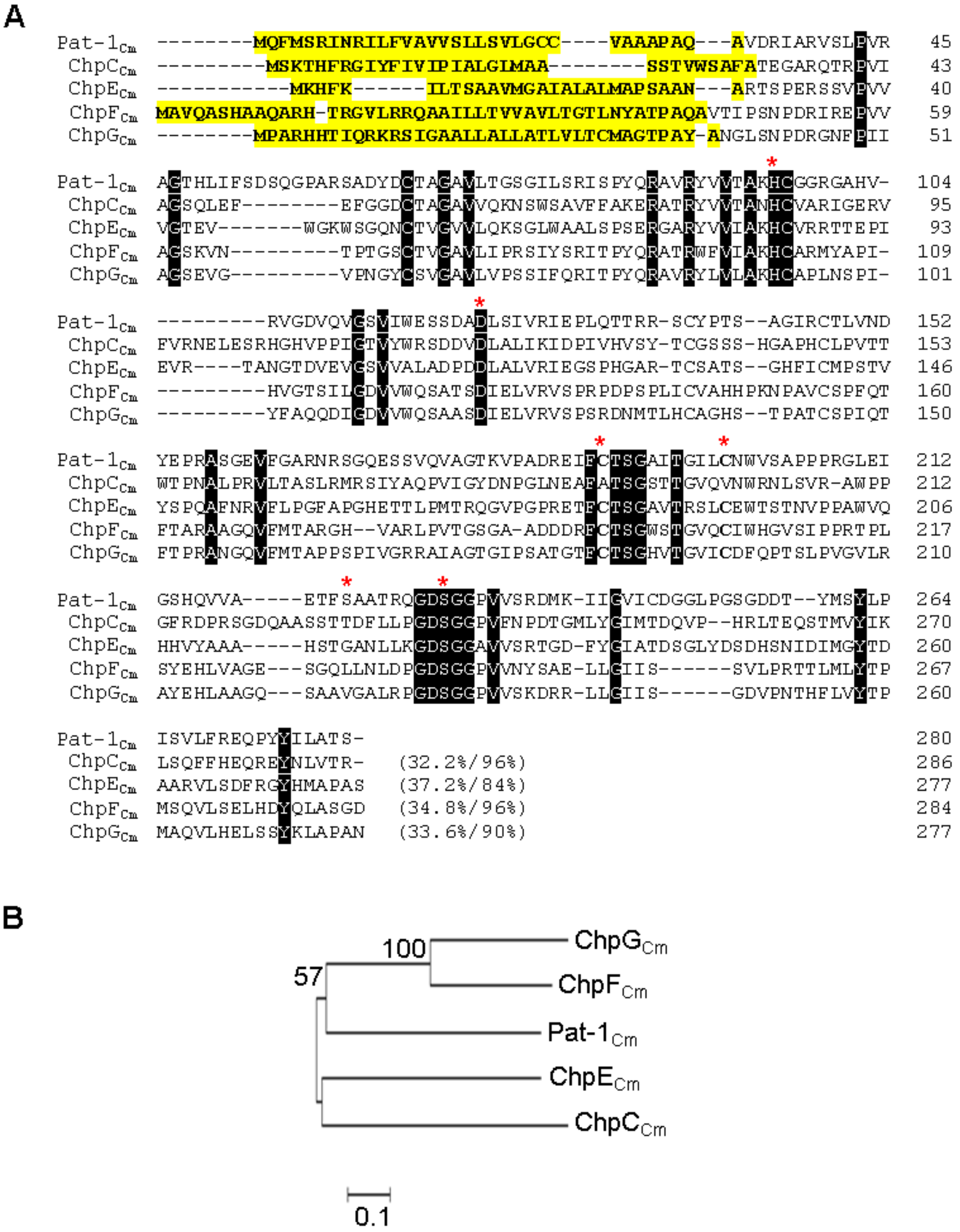

Supplementary Figure 7 Comparison between Pat-1_{Cm} protein and its homologous Chp proteins. **(A)** Amino acid sequence alignment of Pat-1_{Cm} protein with four Chp proteins in *C. michiganensis* shown in Figurs 5 and 7. $ChpA_{Cm}$, $ChpB_{Cm}$ and $ChpG_{Cm}$ were not included because they are pseudo proteins. Conserved amino acid sequences are shown in black or gray. Amino acids highlighted in yellow indicate putative signal peptide predicted with SignalP. * indicates amino acids for catalytic triad and two key cysteines shown in Figure 6. **(B)** Phylogenetic tree of Pat-1_{Cm} protein with four Chp proteins generated with MEGA 6.0.

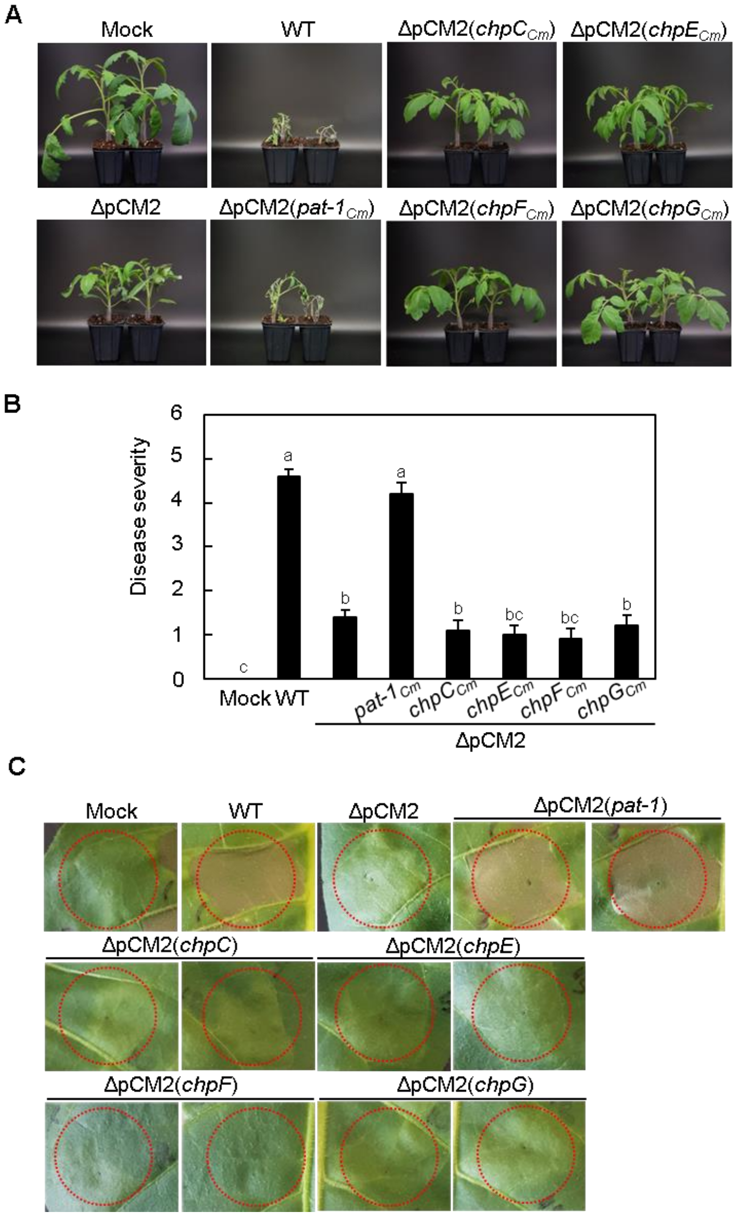

Supplementary Figure 8 No recovery of either pathogenicity or HR-eliciting activities of *C. michiganensis* ΔpCM2 mutant by *chp* genes. **(A)** Wilting symptom development by inoculation of ΔpCM2 mutant carrying each indicated *chp* gene. Inoculated plants were observed for 2 weeks and photographed 14 days after inoculation (dai). **(B)** Disease severity of wilting in tomato plants inoculated with indicated strains at 14 dai. Error bars indicate standard error (n=10). Nonparametric Kruskal–Wallis test with Dunnett's multiple comparisons ($p < 0.05$) was used to analyze the level of disease severity in tomato plants, and different letters indicate statistically significant differences at $p < 0.05$. **(C)** HR phenotype in *N. tabacum* after infiltration with indicated strains. Leaves of 5-week-old plants were inoculated, and representative leaves were photographed 36 h after infiltration. Red dotted lines indicate infiltrated regions. Mock, 10 mM $MgCl_2$; WT, *C. michiganensis* LMG7333 wild type.

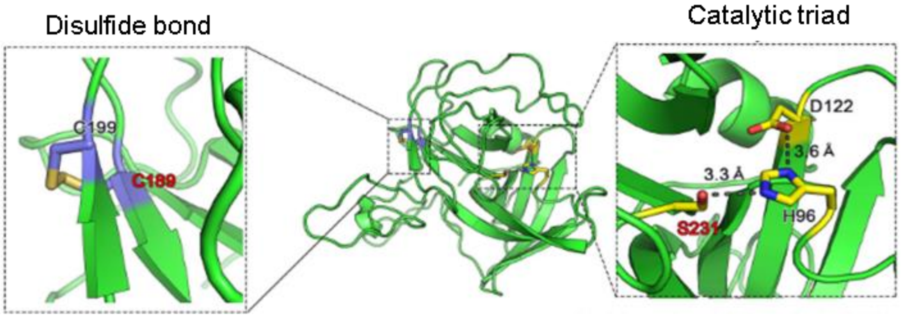

Supplementary Figure 9 Predicted structure of Pat-1_{Cm} protein by homology modeling using the Phyre2. Amino acid residues for putative catalytic triad of enzymatic activity and one disulfide bond for proper folding are magnified in the right and left sides of the predicted structure.

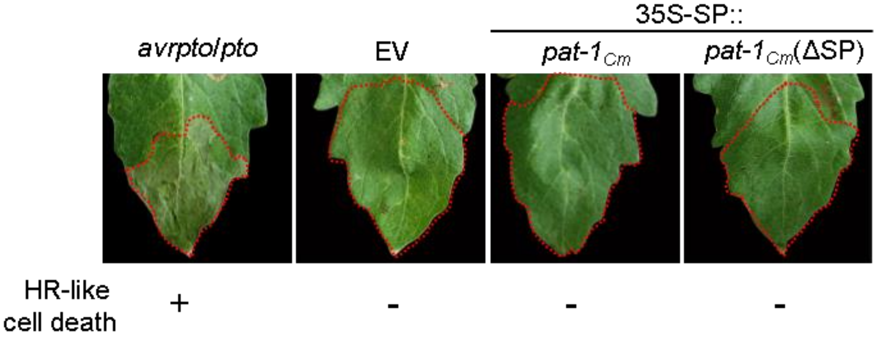

Supplementary Figure 10 No elicitation of HR-like cell death by Pat-1_{Cm} and its variants by *Agrobacterium*-mediated transient expression in tomato. Leaves of 4-week-old tomato plants were infiltrated with *Agrobacterium* carrying each construct for constitutive expression of indicated proteins with the signal peptide (SP) of the tobacco PR1a protein. Red dotted lines indicate the infiltrated regions. Representative leaf was photographed 36 h after infiltration. Empty vector (EV) was used as a negative control.

Supplementary Table 1 Primer list used in this study

Name	Forward primer (5'-3')	Reverse primer (5'-3')
Cm 16S	GTGATGTCAGAGCTTCCTCTGGCGGAT	GTACGGCTACCTTGTTACGACTTAGT
pat-1cm	ATGCAGTTCATGTCGCGCATAA	TCAGGAGGTCGCTAATATGTAA
parA (pCM2)	ATGGAAGCGATCATGGTCT	TCATGAGACAACCTTTGTCA
pat-1cm-OX	ACTAGTAGAACGCTCCCTGCGGCCTTCG	AAGCTTACTTGTCGTCATCGTCTTTGTAGTC GGAGGTCGCTAATATGTAATACGGT
pat-1cc	ATGTCTGTTTACATGTTGCGCATTGGT	AAGCTTACTTGTCGTCATCGTCTTTGTAGTC GGAGGTTGCTAGCACGTAAAACGGTT
chpCcm-OX	ACTAGTCCTATTGACCTGTTCCCCTTGACT	AAGCTTACTTGTCGTCATCGTCTTTGTAGTC ACGTGTCACGAGATTGTATTCTCT
chpEcm-OX	ACTAGTTTCACGTTCGAGGCCTACGCCGAC	AAGCTTACTTGTCGTCATCGTCTTTGTAGTC GCTTGCTGGTGCCATGTGGTATCC
chpFcm-OX	ACTAGTTTTGTCGGCTGAGGCAGTGGAGTG	AAGCTTACTTGTCGTCATCGTCTTTGTAGTC ATCCCCTGAGGCCAATTGATAATC
chpGcm-OX	ACTAGTTTTACCAGTTTCCAGGCCGGTTCA	AAGCTTACTTGTCGTCATCGTCTTTGTAGTC GTTGGCGGGTGCGAGCTTGTAGCT
pat-1cm:pGW417	CACCATGGGATTTTTTCTCTTTTCACAAATG CCCTCATTTTTTCTTGTCTCTACACTTCTCTT ATTCCTAATAATATCTCACTCTTCTCATGCC ATGCAGTTCATGTCGCGCATAAACA	GGAGGTCGCTAATATGTAATACGGT
chpCcm:pGW417	CACCATGGGATTTTTTCTCTTTTCACAAATG CCCTCATTTTTTCTTGTCTCTACACTTCTCTT ATTCCTAATAATATCTCACTCTTCTCATGCC ATGTCAAAAACGCATTTTCGCGGTA	ACGTGTCACGAGATTGTATTCTCTC
chpEcm:pGW417	CACCATGGGATTTTTTCTCTTTTCACAAATG CCCTCATTTTTTCTTGTCTCTACACTTCTCTT ATTCCTAATAATATCTCACTCTTCTCATGCC ATGAAACATTTTAAGATCTTGACGT	GCTTGCTGGTGCCATGTGGTATCCG
chpFcm:pGW417	CACCATGGGATTTTTTCTCTTTTCACAAATG CCCTCATTTTTTCTTGTCTCTACACTTCTCTT ATTCCTAATAATATCTCACTCTTCTCATGCC ATGGCGGTTCAAGCATCGCACGCCG	ATCCCCTGAGGCCAATTGATAATCG
chpGcm:pGW417	CACCATGGGATTTTTTCTCTTTTCACAAATG CCCTCATTTTTTCTTGTCTCTACACTTCTCTT ATTCCTAATAATATCTCACTCTTCTCATGCC ATGCCCGCTCGCCTTCACGCCAT	GTTGGCGGGTGCGAGCTTGTAGCTC

Supplementary Table 2 Putative genes for Tat- and T2SS-secretion systems in *Clavibacter michiganensis* type strain LMG7333

Secretion System	Location No.	Product name	Proteins
Tat	CmLMG7333_1_00841	Sec-independent protein translocase protein TatA	TatA
Tat	CmLMG7333_1_00319	Sec-independent protein translocase protein TatB	TatB
Tat	CmLMG7333_1_00840	Sec-independent protein translocase protein TatC	TatC
Tat	CmLMG7333_1_00514	Signal peptidese I	LepB
Sec	CmLMG7333_1_00185	Protein translocase subunit SecA	*secA*
Sec	CmLMG7333_1_00968	Protein translocase subunit SecD	*secD*
Sec	CmLMG7333_1_01971	Protein translocase subunit SecE	*secE*
Sec	CmLMG7333_1_00967	Protein translocase subunit SecF	*secF*
Sec	CmLMG7333_1_00896	putative protein-export membrane protein SecG	*secG*
Sec	CmLMG7333_1_01773	Protein translocase subunit SecY	*secY*
Sec	CmLMG7333_1_00502	Signal recognition particle receptor FtsY	*ftsY*
Sec	CmLMG7333_1_00503	Signal recognition particle protein	*SRP*
Sec	CmLMG7333_1_00969	hypothetical protein	*yajC*
Sec	CmLMG7333_1_02166	Membrane protein insertase YidC	*yidC*
Sec	CmLMG7333_1_02724	Membrane protein insertase YidC	*yidC*
T2SS	CmLMG7333_1_00440	Type II secretion system protein F	protein F
T2SS	CmLMG7333_1_00441	Type II secretion system F family protein	protein F
